# Small SSEA-4-positive cells from human ovarian cell cultures: related to embryonic stem cells and germinal lineage?

**DOI:** 10.1186/1757-2215-6-24

**Published:** 2013-04-09

**Authors:** Irma Virant-Klun, Martin Stimpfel, Branko Cvjeticanin, Eda Vrtacnik-Bokal, Thomas Skutella

**Affiliations:** 1 Department of Obstetrics and Gynecology, University Medical Centre Ljubljana, Slajmerjeva 3, Ljubljana, 1000, Republic of Slovenia; 2 Institute for Anatomy and Cell Biology, Medical Faculty, University of Heidelberg, Im Neuenheimer Feld 307, Heidelberg, 69120, Germany

**Keywords:** Human, Ovary, Pluripotency, Stem Cells, Primordial germ cells

## Abstract

**Background:**

It has already been found that very small embyronic-like stem cells (VSELs) are present in adult human tissues and organs. The aim of this study was to find if there exists any similar population of cells in cell cultures of reproductive tissues and embryonic stem cells, and if these cells have any relation to pluripotency and germinal lineage.

**Methods and results:**

Here we report that a population of small SSEA-4-positive cells with diameters of up to 4 μm was isolated by fluorescence-activated cell sorting (FACS) from the human ovarian cell cultures after enzymatic degradation of adult cortex tissues. These small cells – putative ovarian stem cells – were also observed during cell culturing of up to 6 months and more. In general, small putative ovarian stem cells, isolated by FACS, showed a relatively low gene expression profile when compared to human embryonic stem cells (hESCs) and human adult fibroblasts; this may reflect the quiescent state of these cells. In spite of that, small putative ovarian stem cells expressed several genes related to primordial germ cells (PGCs), pluripotency and germinal lineage, including *VASA*. The PGC-related gene *PRDM1* was strongly expressed in small putative ovarian stem cells; in both hESCs and fibroblasts it was significantly down-regulated. In addition, putative ovarian stem cells expressed other PGC-related genes, such as *PRDM14* and *DPPA3*. Most of the pluripotency and germinal lineage-related genes were up-regulated in hESCs (except *VASA*). When compared to fibroblasts, there were several pluripotency-related genes, which were up-regulated in small putative ovarian stem cells. Similar populations of small cells were also isolated by FACS from human testicular and hESC cultures.

**Conclusions:**

Our results confirm the potential embryonic-like character of small putative stem cells isolated from human adult ovaries and their possible relation to germinal lineage.

## Introduction

At the beginning it was supposed that pluripotent human embryonic stem cells (hESCs) can be isolated only from the human embryo, but later it was found that embyronic-like stem cells are also present in adult human tissues and organs. The group of Ratajczak first found a novel population of CXCR4+, SSEA-1+, Oct-4+ and CD45- cells in adult bone marrow [1,2] and CXCR4+, SSEA-4+, and Oct-4+ cells in umbilical blood [3]. Because these cells were small and round, with diameters of up to ~5–6 μm, they termed them very small embryonic-like stem cells (VSELs). They hypothesized that these cells are deposited early during development in bone marrow as a mobile pool of circulating pluripotent stem cells that play a crucial role in postnatal tissue turnover and regeneration, both of non-hematopoietic and hematopoietic tissues. Indeed, bone marrow-derived pluripotent VSELs are mobilized into peripheral blood after acute myocardial infarction [4,5], stroke [6], skin burn injury [7], and Crohn’s disease [8]. Adult bone marrow and umbilical cord blood VSELs, expressing the same and other markers of pluripotency (i.e. NANOG, SOX-2), have been confirmed by several other groups [9-15]. Moreover, adult human ovaries offer evidence of cells comparable to VSELs [16-19] being an integral part of the ovarian surface epithelium, namely, and were also proposed for adult human testicles [20-22].

During early development, epiblast/germline-derived cells including primordial germ cells (PGCs) become founder populations of pluripotent stem cells, which are deposited during embryogenesis in various organs and possibly persist as VSELs in these locations into adulthood [23]. Molecular analysis of purified VSELs revealed the expression of several epiblast/PGC markers, migrating PGC-like epigenetic reprogramming profiles of Oct4, Nanog and Stella loci and unique patterns of genomic imprinting [24]. It has been demonstrated that VSELs show hypomethylation or erasure of imprints in paternally methylated genes and hypermethylation of imprints in maternally methylated genes [25]. All these epigenetic characteristics lead to the up-regulation of genes *H19* and *P57 (KIP2* or *CDKN1C*), and repression of *IGF2* and *RASGRF1*. This may explain the VSEL’s quiescent status and lack of teratoma formation in adult tissues and organs. Open chromatin structure of core pluripotency transcription factors supports the pluripotent state of VSELs. Like in other pluripotent stem cells, it has been suggested that VSELs maintain their pluripotent state through an Ezh2-dependent BD-mediated epigenetic mechanism based on genome-wide gene-expression analysis of a small number of highly purified murine bone marrow-derived Oct4-positive VSELs [26].

We were interested to find if there exists any similar population of cells in cell cultures of reproductive tissues and embryonic stem cells, and if these cells have any relation to pluripotency and germinal lineage. We report that small, SSEA-4-positive putative ovarian stem cells were isolated from the ovarian cell cultures. These cells expressed several genes related to pluripotency, embryonic development and germinal lineage, and were similar to those isolated from testicular and embryonic stem cell cultures.

## Material and methods

This study was confirmed by the Slovenian Medical Ethical Committee (Ministry of Health of the Republic of Slovenia, No. 135/09/09 and 154/07/10). Ovarian cortex tissue was surgically retrieved as small biopsies in five patients for different gynecological reasons (non-ovarian cancer, removal of ovaries to prevent breast cancer, myoma or cyst removal). Testicular tissue was retrieved in two infertile men with azoospermia (no sperm in the ejaculate) at diagnostic testicular biopsy to retrieve sperm for in vitro fertilization treatment; in both patients there was no sperm in their testicular tissue due to Sertoli Cell-Only Syndrome (SCOS) or maturation arrest (MA). Each patient’s tissue was used for a research purpose after clear explanation of the study and written consent to participate. One cell culture was established from each ovarian or testicular biopsy. Frozen hESCs of the H1 line (37th passage) were provided from abroad for research purposes only. All cell cultures were established at the University Medical Centre Ljubljana.

### Enzymatic degradation of ovarian cortex or testicular tissues

Ovarian cortex tissue or frozen-thawed testicular tissue (two ampoules per patient) of up to 4 mm^3^, retrieved via biopsy, was enzymatically degraded in the same way. The tissue was transferred into a warm, pre-incubated Dulbecco’s Modified Eagle’s Medium (DMEM)/Nutrient Mixture F-12 Ham with L-glutamine and 15 mM HEPES (D8900; Sigma Aldrich, St. Louis, MO, USA), supplemented with 3.7 g/L NaHCO_3_, 1% penicillin/streptomycin (Sigma Aldrich), and pH adjusted to 7.4 with 1 M NaOH. The whole ovarian/testicular tissue was enzymatically degraded in two steps, as described previously [22]. In the first step it was degraded with collagenase type XI (0.5 mg/mL) and in the second step with collagenase type XI (0.5 mg/mL) and prepared hyaluronidase (SynVitro Hydase; Origio, Måløv, Denmark). After 10 minutes of incubation at 37°C in each enzyme solution, 20% fetal bovine serum (FBS) was added to inactivate enzymes. The suspension of cells was passed through a 70 μm cell strainer (BD Falcon; BD Biosciences, San Jose, CA, USA) and centrifuged for 8 minutes at 1,500 rpm. After centrifugation the supernatant was removed, and the pellets were resuspended in culture medium and placed in the culture dishes.

### Culture of ovarian, testicular and embryonic stem cells

After enzymatic degradation the ovarian or testicular tissue cells were cultured in two different culture media: 1) DMEM/F-12 culture medium with 3.7 g/L NaHCO_3_, 1% penicillin/streptomycin (Sigma Aldrich), pH adjusted to 7.4 with 1 M NaOH and with added 20% (v/v) fetal bovine serum (FBS), on gelatine as the supporting layer or 2) hESC culture medium of DMEM/F-12 medium, including 20% KnockOut Serum Replacement (Gibco Life Technologies, Carlsbad, CA, USA), 1 mM L-glutamine (PAA, Pasching, Austria), 1% nonessential amino acids (PAA), 0.1 mM 2-mercaptoethanol (Invitrogen Life Technologies, Carlsbad, CA, USA), 13 mM HEPES, and 4 ng/mL human basic FGF (Sigma Aldrich), on matrigel as the supporting layer. Ovarian cells and hESCs were cultured in twelve-well culture plates and testicular cells in four-well plates. Cells were cultured in a CO_2_-incubator at 37°C and 6% CO_2_ in air for approximately three months (3.5, 4, 1, 1 and 3.5 months in five ovarian cell cultures and 1 and 3 months in two testicular cultures). In each culture the cells and colonies they formed were monitored daily under a heat-stage equipped inverted microscope at Hoffman or dic-Nomarski illumination and at 200×–6000× magnification (Eclipse T-2000; Nikon, Tokyo, Japan – equipped with a Nikon Digital Sight Camera).

### Immunocytostaining on the expression of surface antigen SSEA-4, nuclear OCT4, SOX-2 and VASA germinal marker

Cells were fixed in 4% paraformaldehyde and permeabilized with 0.2% Triton and then incubated with 3% H_2_O_2_ for 10 minutes to block the endogenous peroxidase activity and for 20 minutes with 10% FBS to block the nonspecific binding sites. Then the cells were incubated for 1 hour at room temperature with rabbit anti-VASA primary antibody (diluted 1:500; Millipore) or mouse anti-SOX2 monoclonal antibody (diluted 1:100, Abcam). After washing with phosphate-buffered saline (PBS), the cells were incubated with biotinylated secondary antibodies, polyclonal goat anti-rabbit immunoglobulins (diluted 1:600; Dakocytomation, Glostrup, Denmark) or polyclonal rabbit anti-mouse immunoglobulins (diluted 1:400; Dakocytomation), for 30 minutes and then with an ABC reagent (Vectastain ABC Kit-Standard; Vector Laboratories, Burlingame, CA, USA) for 30 minutes. Finally, the cells were incubated in a DAB substrate (Sigma Aldrich) until brown staining appeared (but no longer than 5 minutes), washed with PBS and observed under an inverted microscope (Hoffman illumination) to detect positive brown-stained cells or cell colonies. Immunofluorescence was used to detect the expression of OCT4 and also SSEA-4. Briefly, cells were fixed in 4% paraformaldehyde, permeabilized with 0.2% Triton (for OCT4) and incubated for 20 minutes with 10% FBS. Then the cells were incubated for 1 hour with polyclonal rabbit anti-OCT4 primary antibodies (diluted 1:100; Stemgent, Boston, MA, USA) or mouse anti-stage-specific embryonic antigen-4 (SSEA-4) monoclonal antibody (clone MC-813-70, diluted 1:100; Millipore, MA, USA) and after washing, again for 30 minutes with Cy3 goat anti-rabbit secondary antibodies (diluted 1:200; Invitrogen) or with anti-mouse immunoglobulins conjugated to Alexa Fluor 488 (1:200; Molecular Probes). After washing the cells were mounted using Vectashield mounting medium with DAPI and observed under a fluorescence microscope. For a negative control, the primary antibodies were omitted from the procedure and replaced with 1% FBS.

### Alkaline phosphatase staining

To detect alkaline phosphatase (AP) activity an Alkaline Phosphatase Detection Kit (Millipore) was used. Cells were fixed in 4% paraformaldehyde for 3 minutes and then incubated in a working solution of reagents which consisted of Fast Red Violet, Naphtol AS-BI phosphate solution and water in a 2:1:1 ratio. After 15 minutes the cells were rinsed with PBS and observed under an inverted microscope (Hoffman illumination). The cells or cell colonies expressing AP activity were stained pink to red.

### Fluorescence-activated cell sorting (FACS)

Cells from one twelve-well plate of each culture were trypsinized (0.15% solution of trypsin-EDTA) and sorted based on SSEA-4 expression. The SSEA-4-positive cells were isolated from a cell suspension of each cell culture using fluorescence-activated cell sorting (FACSAria, BD Biosciences, San Jose, CA, USA). Briefly, 10^6^ cells were resuspended in PBS with 1% penicillin/streptomycin (Sigma Aldrich) and stained with anti-SSEA-4 antibodies (clone MC813-70; BD Pharmingen, San Jose, CA, USA) conjugated with phycoerythrin (PE). The sample stained with an appropriate isotype control (PE Mouse IgG3, κ; BD Pharmingen) was examined in parallel. All antibodies were added at saturation concentrations, and the cells were incubated for 30 minutes in the dark, then washed and resuspended for sorting in PBS with penicillin/streptomycin at a concentration of 2 × 10^6^ cells/ml. Sorted cells were collected in Dulbecco’s Modified Eagle’s Medium (DMEM)/Nutrient Mixture F-12 Ham with L-glutamine and 15 mM HEPES (D8900; Sigma Aldrich), supplemented with 3.7 g/L NaHCO_3_ (S5761; Sigma Aldrich), 1% penicillin/streptomycin, 0.5% gentamycin (G1272; Sigma Aldrich) and pH adjusted to 7.4 with 1 M NaOH. After FACS cells were cultured in DMEM/F-12 culture medium with 20% fetal bovine serum. Droplets of sorted cells were DAPI stained and monitored under a fluorescence microscope.

### Gene expression analyses by microarrays

Three samples of populations of SSEA-4-positive putative ovarian stem cells (OSC), isolated by FACS from three different ovarian cell cultures in three different patients were analyzed by microarrays and compared with positive control – three samples of hESCs (ESC) of H1 cell line (WiCell, WI, USA), and negative control – three samples of human adult (dermal) fibroblasts (FB). Samples were lysed using SuperAmp™ Lysis Buffer and stored at -20°C. When collected, the samples were analyzed by Miltenyi Biotec (Bergisch Gladbach, Germany) according to established protocols. First the SuperAmp RNA amplification was performed. Amplified cDNA samples were quantified using the ND-1000 Spectrofotometer (NanoDrop Technologies, Wilmington, DE, USA). The integrity of cDNA was checked via the Agilent 2100 Bioanalyzer platform (Agilent Technologies, Wokingham, UK). The Cy3-labeled cDNAs were hybridized overnight to an Agilent Whole Human Genome Oligo Microarrays 8×60K and washed. Fluorescence signals of the hybridized Agilent Microarrays were detected using Agilent’s Microarray Scanner System (Agilent Technologies). The Agilent Feature Extraction Software was used to read out and process the microarray image files.

### Statistics of microarray analyses

Two differential gene expression analyses (DGAs) were performed: hESCs vs. OSCs (FACS-isolated SSEA-4-positive small putative ovarian stem cells) and fibroblasts vs. OSCs (FACS-isolated SSEA4-positive small putative ovarian stem cells) to identify differently expressed transcripts. For determination of differential gene expression (DGA) derived output data files were further analyzed using the Rosetta Resolver gene expression data analysis system (Rosetta Biosoftware). Data were preprocessed by normalization and correlation analysis. The normalized intensities were log2-transformed and served as basis for further analysis. Heatmaps were created based on ratio data in log2 space to get a more intuitive visual inspection of gene expression data. A Student’s t-test was performed on each gene separately using normalized log2 intensity data. Statistical significance was set at p < 0.05. The genes selected as reliable candidates for differentially expressed genes were required to show at least a 16-fold average expression difference (log ratio = 4) between the sample groups. Due to some variability, a more stringent selection was performed for the individual comparisons to get a statistically high confidence list of differently expressed genes. To elucidate the gene functions and pathways IPA Software was used (Ingenuity Systems, Redwood City, CA, USA). We focused on genes related to primordial germ cells, pluripotency and germinal lineage, and genes up-regulated or down-regulated in putative ovarian stem cells in comparison with other groups of cells (hESCs and fibroblasts).

### RT-PCR analyses

The samples remained after microarray analyses were analyzed by RT-PCR. Gene expression analyses of three samples of putative ovarian stem cells (OSC1-3) were performed using the Biomark Real-Time quantitative PCR (qPCR) system (Fluidigm, San Francisco, CA, USA). In all samples, expressions of 7 genes: *LEFTY1*, *SALL4*, *OCT4A*, *TDGF1*, *REC8*, *CDH1* and *STELLA*, related to pluripotency and embryonic stem cells, and of the housekeeping gene *GAPDH*, which was used for normalization, were analyzed in comparisons with three samples of positive control – hESCs (ESC1-3), and three samples of negative control – human adult (dermal) fibroblasts (FB1-3). The inventoried TaqMan assays (20×, Applied Biosystems, Carlsbad, CA, USA) were pooled to a final concentration of 0.2× for each of the 8 assays. The samples were put directly into 9 μL RT-PreAmp Master Mix (5.0 μL CellsDirect 2× Reaction Mix (Invitrogen); 2.5 μL 0.2× assay pool; 0.2 μL RT/Taq Superscript III (Invitrogen); 1.3 μL TE buffer). The sequencespecific reverse transcription was performed at 50°C for 15 minutes. The reverse transcriptase was inactivated by heating to 95°C for 2 minutes. Subsequently, in the same tube cDNA went through limited sequence-specific amplification by denaturing at 95°C for 15 seconds, and annealing and amplification at 60°C for 4 minutes for 14 cycles. These pre-amplified products were diluted 5-fold prior to analysis with Universal PCR Master Mix and the inventoried TaqMan gene expression assays (ABI) in 96.96 Dynamic Arrays on a BioMark System. Each sample was analyzed in two technical replicates. Ct values were obtained from the BioMark System and were transferred to the GenEx software (MultiD, Göteborg, Sweden). The compared groups of samples were analyzed on heatmap and hierarchical clusterings (Ward’s Algorithm, Euclidean Distance Measure) and by principal component analysis (PCA). In addition, descriptive statistics were calculated individually for the genes using a 0.95% confidence level and groups of cells (OSCs vs. hESCs and OSCs vs. fibroblasts FBs) were compared by an unpaired 2-tailed Student’s T-test; to keep the overall risk of error at 0.05 a threshold value of 0.00730 was used according to the Dunn-Bonferoni correction.

## Results

**Figure 1:**
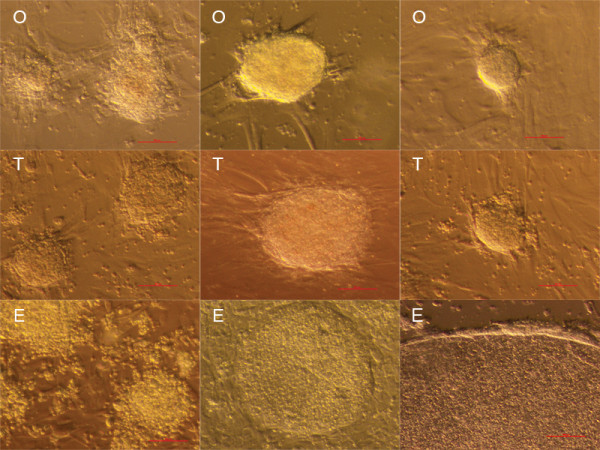
**Cell colonies formed in ovarian (O), testicular (T) and human embryonic stem cell (E) cultures under inverted microscope.** Scale bar: 50 μm.

**Figure 2:**
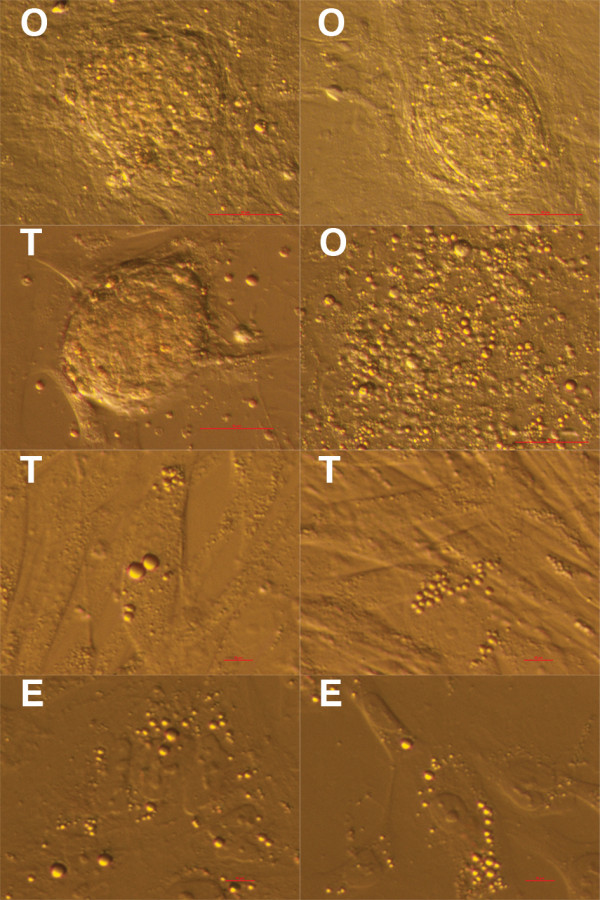
**Small, round, yellow-coloured cells with diameters of up to 4 μm – putative stem cells – forming cell colonies or appearing as single cells.** In ovarian (**O**), testicular (**T**) and early human embryonic stem (**E**) cell cultures after thawing, under an inverted microscope. Scale bars: 50 μm and 10 μm.

In all cell cultures comparable cell colonies were developed (Figure 1). Cell colonies in ovarian and testicular cell cultures were similar, and were quite comparable to early cell colonies in hESC cultures. During culturing in all cell cultures, small, round, yellow-coloured cells with diameters of up to 4 μm and with very similar morphology were observed among other cell types (Figure 2). These cells were not blood cells or cells from the immunological system, as previously confirmed by May-Grünwald-Giemsa staining [18]. Also, they were not lipid droplets, because they did not stain by Oil Red O (data not shown).

**Figure 3:**
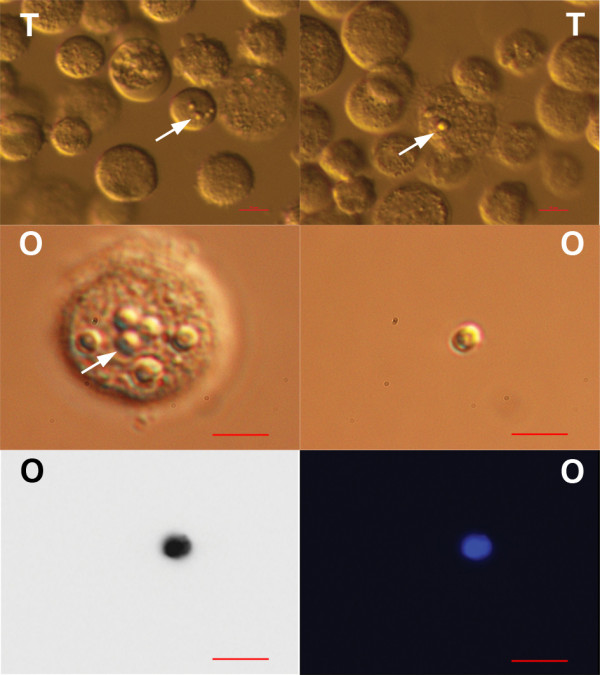
**Small, round, yellow-coloured cells (arrows) – putative stem cells – with diameters of up to 4 μm: from testicular (T) cell cultures (attached to germinal cells, Sertoli cells or fibroblasts) or from ovarian (O) cell cultures (attached to epithelial or other types of cells).** DAPI (blue) stained nuclei from ovarian (**O**) cell cultures. Scale bars: 10 μm.

**Figure 4:**
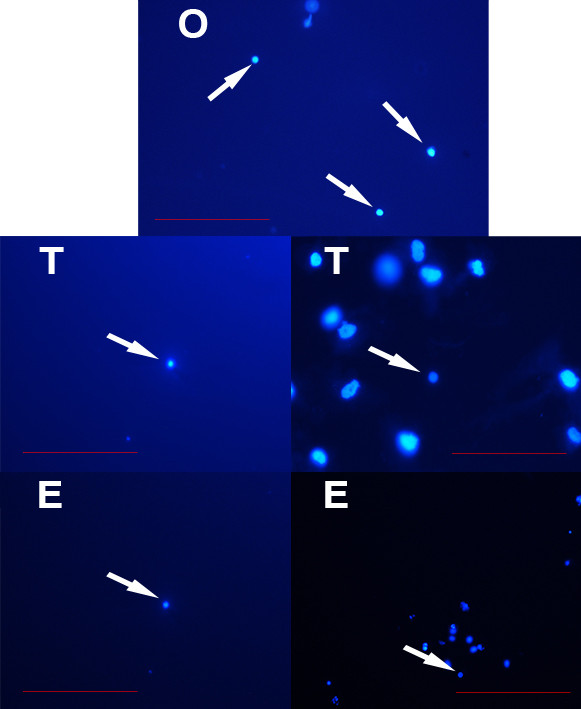
**DAPI stained (blue) nuclei (arrows) of small putative stem cells from ovarian (O), testicular (T) and human embryonic stem (E) cell cultures.** Scale bars: 100 μm

In ovarian and testicular cell cultures these small, round, yellow-coloured cells were more abundant when cultured in hESC culture medium than in DMEM/F-12 culture medium with added FBS. They were appearing throughout the entire cell culturing process. At some places it was clearly seen that they formed cell colonies or were present around them (Figure 2). These small cells were appearing as single cells or were attached to other types of cells (Figure 3). They showed a high affinity to other types of cells (e.g. fibroblasts, epithelial cells) and were mostly attached to them. In hESC cultures these cells were more abundant in early cell cultures, establishing after thawing the cells, but were also present in an established cell culture with typical, well-developed cell colonies. In testicular stem cell cultures these small round cells were attached to different male germinal cells (e.g. spermatogonia, spermatocytes, round spermatids), Sertoli cells or fibroblasts (Figure 3). In ovarian cell cultures they were also attached to bigger cells (e.g. epithelial cells, fibroblasts) (Figure 3). After DAPI staining these small cells exhibited high nucleus/cytoplasm ratios (Figures 3 and 4).

### SSEA-4-positive cells sorted by FACS

**Figure 5:**
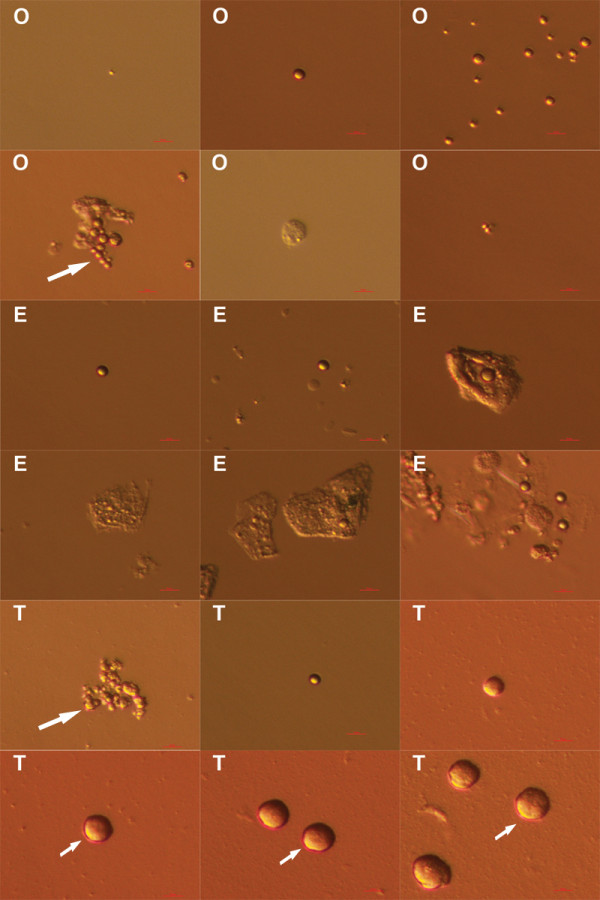
**SSEA-4-positive cells – putative stem cells – isolated by fluorescence-activated cell sorting (FACS) from ovarian (O), testicular (T) and human embryonic stem (E) cell cultures were small, round, yellow-coloured cells with diameters of up to 4 μm.** They appeared as single cells or were attached to bigger cells. In one ovarian (**O**) cell culture and one (**T**) testicular cell culture these small cells were proliferating (long arrows) or growing into bigger round cells (short arrows) with diameters of up to 10 μm. Scale bars: 10 μm.

**Figure 6:**
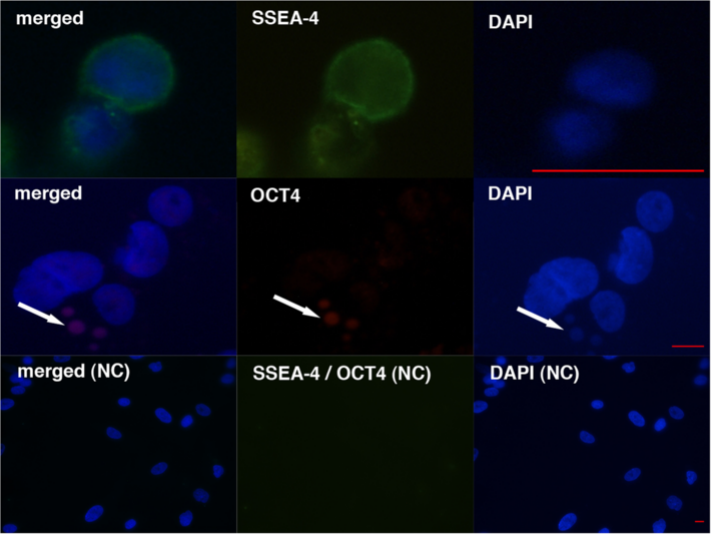
**Small round cells – putative stem cells – in ovarian cell cultures were SSEA-4 and OCT4-positive (arrows) after immunofluorescence staining. Merged, SSEA-4 (green), OCT4 (red) and DAPI (blue) stainings are presented for samples and for a negative control (NC).** Scale bars: 10 μm.

**Figure 7:**
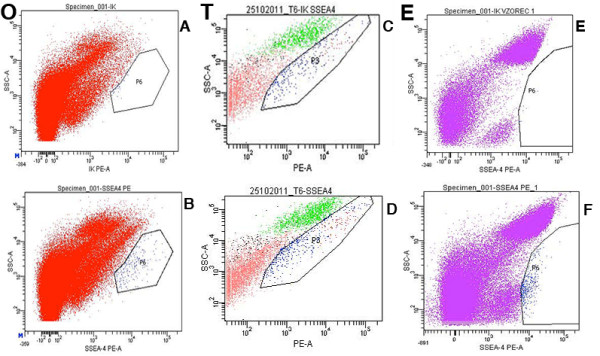
**Fluorescence-activated cell sorting of SSEA-4-positive small putative stem cells from the ovarian (O), testicular (T) and human embryonic stem (E) cell cultures.** Sorted cells are in the marked frames. (**A, C, E**) Isotype controls. (**B, D, F**) Samples.

The mean duration of cell cultures at the time of FACS was 2.6 months (min. 1 – max. 4) for ovarian cell cultures and 2 months (1 month and 3 months) for testicular cell cultures. After trypsinisation of ovarian, testicular and embryonic stem cell cultures, a very similar population of SSEA-4-positive cells was isolated from each culture by FACS, as can be seen in Figure 5. These cells were predominately small, round, yellow-coloured cells with diameters of up to 4 μm. Like during culturing, these cells were appearing as single cells or were attached to bigger cells (Figure 5). When observed after immunofluorescence staining, these cells were confirmed to be SSEA-4-positive (Figure 6). The expression of surface antigen SSEA-4, an established marker of pluripotency [27], makes them putative stem cells. In one testicular and one hESC culture some of the sorted small, round, yellow-coloured cells – putative stem cells – grew and developed into cells with diameters of up to 10 μm (Figure 5). The SSEA-4-positive cells represented 2.2% of all cells on average when sorted from the ovarian cell cultures, 1.4% of cells on average when sorted from the testicular cell cultures and 0.8% of cells when sorted from hESC cultures. The population of putative stem cells sorted from the hESC culture was very similar to those sorted from reproductive tissues, as can be seen in Figure 5. The SSEA-4-positive small, round cells – putative stem cells – sorted by FACS (Figure 7) were quite comparable to VSELs in terms of their morphology and diameters.

### Positivity for OCT4, SOX-2 and alkaline phosphatase activity

**Figure 8:**
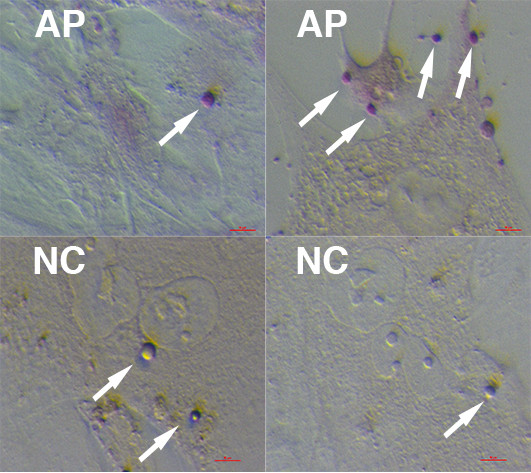
**Small putative stem cells in ovarian and testicular cell cultures were positively stained (arrows) on alkaline phosphatase (AP).** Scale bars: 10 μm. *Legend*: AP (red) – alkaline phosphatase activity; NC – negative control.

**Figure 9:**
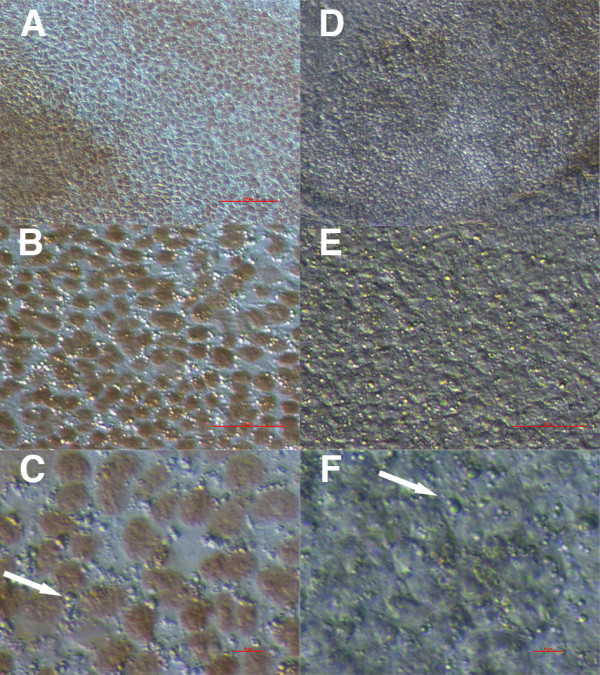
**Small putative stem cells (arrows) in ovarian and testicular cell cultures were positively stained on alkaline phosphatase (AP).** A proportion of small putative stem cells were SOX-2 positive. Scale bars: 100 μm (**A**, **D**); 50 μm (**B**, **E**); 10 μm (**C**, **F**).

When in the ovarian cell culture, these small cells were OCT4-positive, as revealed by immunofluorescence (Figure 6) and were positively stained on alkaline phosphatase (AP) acitivity (Figure 8). In addition, these small cells were present in a hESC culture with typical, well-developed cell colonies (Figure 9). After immunocytostaining for the SOX-2 marker of pluripotency, a proportion of these small putative stem cells were positively stained (Figure 9).

### VASA-positivity of cell cultures

**Figure 10:**
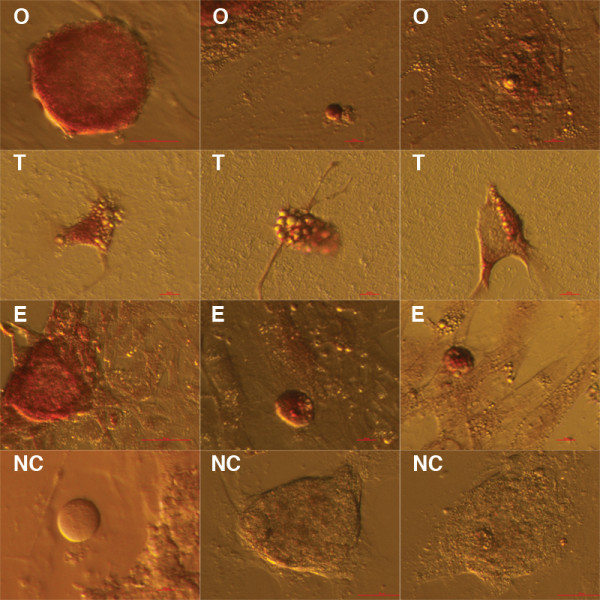
**A proportion of cell colonies and small putative stem cells in ovarian (O), testicular (T) and human embryonic stem (E) cell cultures were VASA-positive in comparison with negative controls (NC) under an inverted microscope.** Scale bars: 50 μm and 10 μm.

Surprisingly, a low proportion of the small round cells – putative stem cells – and only some of the cell colonies formed in all cell cultures, including hESCs, were VASA-positive (Figure 10), as revealed by immunocytochemistry, thus showing the possible relation to the germ lineage.

### Microarray analyses of small putative ovarian stem cells in comparison with hESCs and human adult fibroblasts

#### General gene expression profile

**Figure 11:**
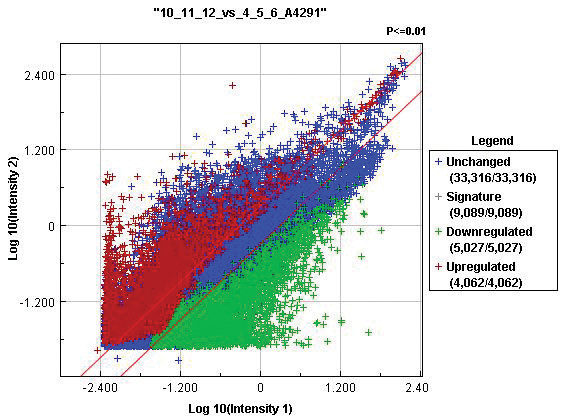
**Gene expression profile of human embryonic stem cells (10_11_12) in comparison with FACS-sorted SSEA-4-positive small putative ovarian stem cells (4_5_6).** The signal intensities of each feature represented by a dot are shown in a double logarithmic scale. X-axis: control-log signal intensity; Y-axis: sample-log signal intensity. *Legend*: Red diagonal lines – the areas of 2-fold differential signal intensities; Blue cross – unchanged genes; Red cross – significantly up-regulated genes (p-value < 0.01); Green cross – significantly down-regulated genes (p-value < 0.01); Grey cross – summary of significantly up- and down-regulated signatures.

When hESCs were compared to FACS-sorted SSEA-4-positive putative ovarian stem cells with diameters of up to 4 μm by Agilent Whole Genome Oligo Microarrays, it was confirmed that 33,316 genes were unchanged, 5,027 genes were down-regulated and 4,062 genes were up-regulated in hESCs (Figure 11). The comparison of fibroblasts and ovarian stem cells showed a higher difference: 27,182 genes were unchanged, 7,149 genes were down-regulated and 8,074 genes were up-regulated in fibroblasts. In spite of the comparable cDNA concentrations to be analyzed (hESCs: 174.4, 175.6 and 208.5 ng/μL; putative ovarian stem cells: 255.1, 180.2 and 189.5 ng/μL; fibroblasts: 232.0, 254.9, and 206.2 ng/μL), the overall intensity distribution of samples with SSEA-4-positive putative ovarian stem cells was lower than for hESCs and fibroblasts and may reflect a more quiescent state.

#### Expression of genes related to pluripotency

**Figure 12:**
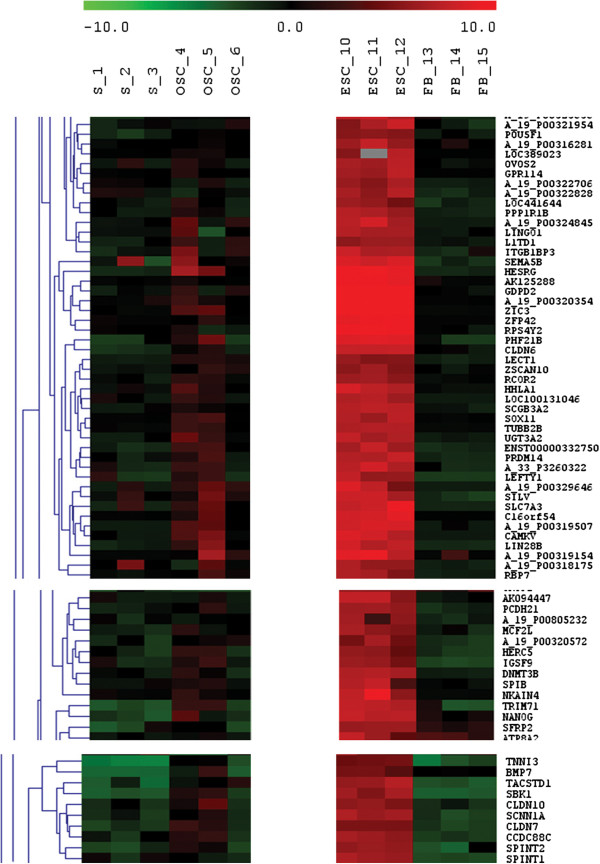
Heatmap expressions (excerpt) of genes up-regulated in human embryonic stem cells (hESCs), including genes related to pluripotency, in comparison with FACS-sorted small putative ovarian stem cells (OSCs), with non-sorted ovarian cell cultures, released from the culture dish bottom with trypsinisation (S) and with human adult fibroblasts (FBs).

**Figure 13:**
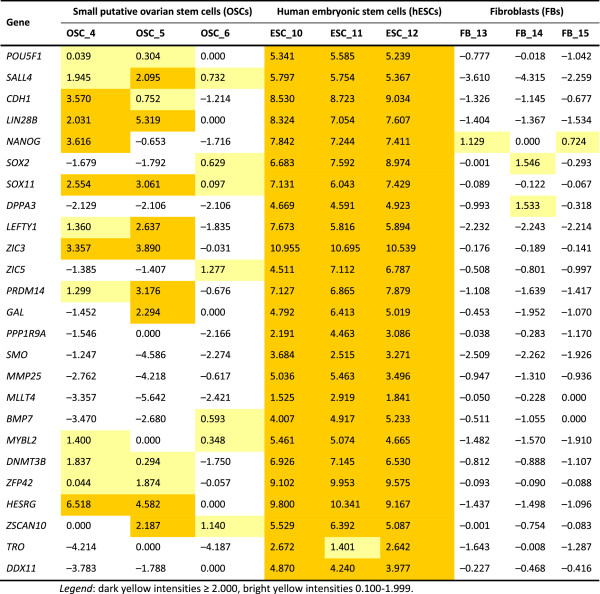
The expressions of genes (normalized and log2-transformed intensities) related to pluripotency, embryonic stem cells and embryogenesis in FACS-isolated small putative ovarian stem cells (OSCs) in comparison with human embryonic stem cells (hESCs) and fibroblasts (FBs).

The FACS-sorted SSEA-4-positive small putative ovarian stem cells expressed several genes related to pluripotency, cell self-renewal, embryonic development and implantation, such as *POU5F1* (*OCT4*), *SALL4*, *CDH1*, *LIN28B*, *NANOG*, *SOX2*, *SOX11*, *DPPA3* (*STELLA*), *LEFTY1*, *ZIC3*, *ZIC5*, *PRDM14*, *GAL*, *PPP1R9A*, *RNF2*, *LASS1* (*CERS1*), *SMO*, *MMP25*, *GULP1*, *MLLT4*, *BMP7*, *MYBL2*, *DNMT3B*, *ZFP42*, *HESRG*, *ZSCAN10*, *TRO*, *GLI2*, *FBN3* and *DDX11*. In comparison to hESCs, all these genes were up-regulated in hESCs, as can be seen in Figures 12 and 13, Additional file 1: Table S1. The most expressed pluripotency and embryogenesis-related genes in hESCs were *ZIC3*, *ZFP42* and *HESRG* and in small putative ovarian stem cells *HESRG*, *ZIC3* and *LIN28B*. The three samples of FACS-isolated SSEA-4-positive small putative ovarian stem cells were heterogenous; two samples more strongly expressed the genes related to pluripotency than the third sample, as can be seen in Figures 12 and 13. On the other hand, these genes were not expressed or were only weakly expressed in human adult fibroblasts (Figure 13). When these two samples of small putative ovarian stem cells with more expressed pluripotency were compared to fibroblasts, there were several pluripotency and embryogenesis-related genes which were with statistical significance up-regulated in small putative ovarian stem cells, such as *SALL4*, *CDH1*, *LIN28B*, *SOX11*, *LEFTY1*, *ZIC3*, *PRDM14*, *PPP1R9A*, *MYBL2*, *DNMT3B* and *HESRG* (Table 1). On the other hand, there was one gene – *MMP25* – down-regulated in small putative ovarian stem cells. The expression of genes related to pluripotency was not detected in the whole trypsinized ovarian cell culture, which included an abundant autologous ovarian fibroblast layer (Figure 12).

**Table 1:** Differences in the expressions of genes (normalized and log2-transformed intensities) related to pluripotency, embryonic stem cells and embryogenesis in FACS-isolated small putative ovarian stem cells (OSCs) in comparison with fibroblasts (FBs) revealed using the Student’s T test

	**Log fold change**	**p-value**	**Adj. p-value**
*POU5F1*	0.78	0.122520121	1.64E-01
SALL4 **	5.41	0.000714457	2.23E-03
CDH1 **	3.21	0.018838123	3.21E-02
LIN28B **	5.11	0.005075958	1.06E-02
*NANOG*	0.86	0.552979106	6.08E-01
*SOX2*	−2.15	0.023394118	3.87E-02
SOX11 **	2.90	0.000290721	1.18E-03
*DPPA3*	−2.19	0.048897088	7.36E-02
LEFTY1 **	4.23	0.000384105	1.42E-03
ZIC3 **	3.79	9.29E-05	5.66E-04
*ZIC5*	−0.63	0.090116973	1.25E-01
PRDM14 **	3.63	0.003039563	6.94E-03
*GAL*	1.58	0.26269284	3.19E-01
PPP1R9A **	1.55	0.006663692	1.33E-02
*SMO*	−0.68	0.545353659	6.01E-01
MMP25 *	−2.43	0.006783372	1.35E-02
*MLLT4*	−1.10	0.211498682	2.64E-01
*BMP7*	0.73	0.515756119	5.73E-01
MYBL2 **	2.35	0.006965786	1.38E-02
DNMT3B **	2.64	0.000957838	2.79E-03
*ZFP42*	1.05	0.148285014	1.94E-01
HESRG **	6.89	0.000200358	9.14E-04
*ZSCAN10*	1.37	0.130067706	1.73E-01
*TRO*	−1.13	0.456996609	5.17E-01
*DDX11*	−2.57	0.015893686	2.78E-02

P-values were adjusted for the effect of multiple testing according to Benjamini-Hochberg (limma, Bioconductor, programme R 2.14.1). Statistical significance was set at p < 0.05. *Legend*: ** – genes up-regulated in OSCs, * – genes down-regulated in OSCs.

#### Expression of genes related to primordial germ cells

Microarray analysis showed that the primordial germ cell (PGC)-related gene *PRDM1* (*BLIMP1*) was strongly expressed in small putative ovarian stem cells. In both hESCs (p = 8.29E-03) and human adult fibroblasts (p = 2.71E-04) *PRDM1* was significantly down-regulated at the log ratio < −4 in comparison with ovarian stem cells, as revealed by DGAs. In addition putative ovarian stem cells expressed *PRDM14*, but the expression of this gene was up-regulated in hESCs (p = 6.41E-03) and down-regulated in fibroblasts (p = 6.94E-03). The *DPPA3* (*STELLA*) gene was expressed in small putative ovarian stem cells, but it was significantly up-regulated in hESCs (p = 2.79E-07).

#### Expression of genes related to germinal lineage

In the FACS-sorted SSEA-4-positive small putative ovarian stem cells and hESCs several genes related to germinal lineage were expressed. When both groups of cells were compared, some germinal lineage-related genes, such as *DPPA3* (*STELLA*), *KIT*, *DNMT3B*, *MSH4*, *BNC2*, *DIAPH2* and *NR6A1* were up-regulated in hESCs. On the other hand, some other germinal lineage-related genes, such as *VASA* (*DDX4*) and *PLD6* were down-regulated in hESCs in comparison with small putative ovarian stem cells, as can be seen in Additional file 1: Table S2. These genes were not expressed or were weakly expressed in adult human fibroblasts. In fibroblasts transcription factor *OVOL1*, critical for oogenesis and spermatogenesis was expressed, but was at a statistical high confidence down-regulated in comparison with FACS-sorted SSEA-4-positive small putative ovarian stem cells (fold change −32.760, p = 2.92E-03).

#### Genes down-regulated in hESCs in comparison with small putative ovarian stem cells

According to the DGA (hESCs vs. OSCs) there were 341 genes, which were with high statistical confidence down-regulated in hESCs when compared to FACS-sorted SSEA-4-positive small putative ovarian stem cells (OSCs). When the character of these genes was inspected, down-regulation of imprinted maternally expressed *H19* was found and at least 75 other genes were found to be related to oncogenesis (e.g. *S100P*, *NOX4*, *WNT5B*, *BRIP1*, *NRP2*, *CHD13*, *CPA4*, *ABLIM3*, *MGLL, FGF7*), tumor suppression or reduction of metastatic potential (e.g. *MPP1*, *SERPINB8*, *PML*, *DLEU2L*, *MXI1*, *YAP1*, *INTS6*, *GPNMB*, *CAV2*), transcription regulation (e.g., *PITX1*, *HOXA11*, *FOXC1*, *PML*, *FOXO4, ZBTB26*, *HMBOX1*, *ZNF41*, *PHTF2*, *MXI1*, *YAP1*, *MBD2*, *ZNF518B*), embryogenesis (e.g. *WNT5B*, *FOXC1*, *PML*, *ABLIM3*, *ACVR1*, *FGF7, IL6ST*), cellular processes such as cell survival/death (apoptosis), proliferation, division, differentiation and cell cycle progression (e.g. *TGM2*, *S100P*, *ARHGDIB*, *NOX4*, *MPP1*, *WNT5B*, *SPDYA*, *CDK8*, *SERPINB8*, *PML*, *DAAM1*, *FOXO4*, *TGFB2*, *CFLAR*, *CCNY*, *CSNK1G3*, *DYRK4*, *PARVA*, *YAP1*, *ZNF703*, *SEPT8*, *CACNA1C*, *FGF7*, *GPNMB*, *CAV2, PPH1F*), inactivation of chromosome X (*XIST*), morphogenesis (*HOXD9*, *FRY*, *FGF7*, *UACA*), left-right organ asymmetry (*PITH1*) and tissue repair (*LUM*). Among genes related to development of tissues and organs, the genes involved in neural development (e.g. *FRMPD4*, *PITX1*, *FRY*, *MPP1*, *SMARCA2*, *PML*, *CDH13*, *FAM114A1*, *SPON2*, *PLXNB3*, *PCDHGB4*, *ZNF703*, *SEMA5A*, *NRP2*) predominated, followed by genes related to the development of the cardiovascular system (e.g. *PECAM1*, *NRP2*, *ANGPT1*, *FBLN5,* ANGPT1), ovaries (*FOXL2*), endometrium (*HOXA11*), lungs (*FGF7*), myoblasts (*PPAPDC3*), lymphocytes (e.g. *NT5E*, *MR1, STAM*) and development of hematopoiesis (*RUNX1*). The character of these genes might be related to the degree of stemness. Several of these genes were down-regulated or were not expressed in human adult fibroblasts. The locations and functions of genes down-regulated in hESCs in comparison with putative ovarian stem cells are described in Additional file 1: Table S3.

#### Genes up-regulated in hESCs in comparison with small putative ovarian stem cells

On the other hand there were 435 genes which were at a high statistical confidence up-regulated in hESCs in comparison with FACS-sorted SSEA-4-positive small putative ovarian stem cells (OSCs), as revealed by DGA analysis (hESCs vs. OSCs). Among these genes there were several genes related to pluripotency, cell self-renewal, embryonic development and implantation, as already described above (Figure 12). The locations and functions of genes up-regulated in hESCs in comparison with small putative ovarian stem cells are described in the Additional file 1: Table S4. Among other genes, some imprinted genes, such as *SNRPN*, *PPP1R9A* and *DNMT3B* were up-regulated in hESCs. From the heatmap presenting the expression of genes up-regulated in hESCs it can be seen that the FACS-sorted SSEA-4-positive small putative ovarian stem cells significantly expressed several genes related to pluripotency and embryogenesis, while human adult fibroblasts did not express or weakly expressed most of genes, which were up-regulated in hESCs (Figure 12).

#### Comparison of fibroblasts to small putative ovarian stem cells

**Figure 14:**
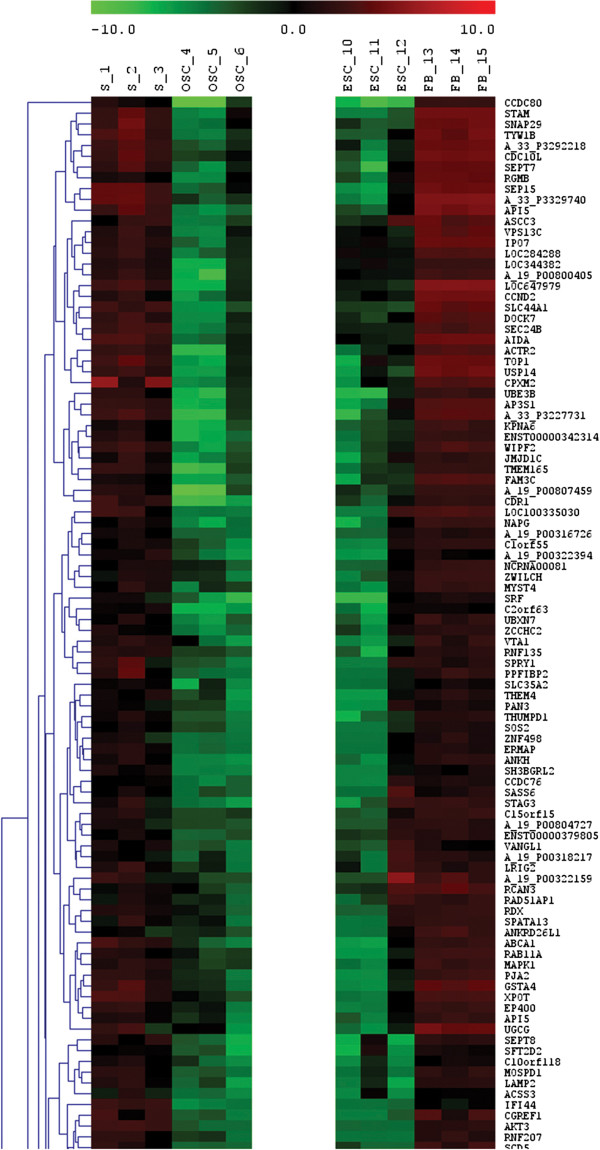
Heatmap expressions (excerpt) of genes up-regulated in human adult fibroblasts (FBs) in comparison with FACS-sorted small putative ovarian stem cells (OSCs), human embryonic stem cells (hESCs) and with non-sorted ovarian stem cell cultures, released from the culture dish bottom with trypsinisation (S).

When human adult fibroblasts were compared to small putative ovarian stem cells, the proportion of differently expressed genes was significantly higher than in hESCs. Fibroblasts had 239 down-regulated genes and 1,084 up-regulated genes in comparison with FACS-isolated SSEA-4-positive small putative ovarian stem cells, as revealed by DGA analysis (FBs vs. OSCs). In fibroblasts the gene *PROM1* encoding the surface antigen CD133 was significantly down-regulated (p = 3.78E-02) at the log ratio < −4 in comparison with ovarian stem cells, as revealed by DGA. Moreover, in fibroblasts the imprinted maternally expressed *H19* and paternally expressed *IGF2* were down-regulated. As can be seen in Figures 12 and 13, the fibroblasts did not express or only weakly expressed a variety of genes related to pluripotency. There were several genes related to pluripotency and embryogenesis (e.g. *PTX1*, *SALL4*, *DPPA5*, *ELF3*, *FGF9*, the *SOX*-family of genes: *SOX3*, *SOX6*, *SOX14*, *SOX15*, *SOX30*), morphogenesis (e.g. *HOXD11*, *FGF9*), cell cycle and cell survival regulation (*CDCA3*, *CENPM*, *DTL*, *NOX4*, *E2F2*), transcriptional regulation (*ZNF114*, *E2F2*, *ELF3*, *HOXD11*, *OVOL1*), cell growth, differentiation and development (*IGF2*, *FGF9*, *OVOL1*, *CGA*, *ESRRB*, *E2F2*), tumorigenesis/cancer (*ELF3*, *NOX4*, *FGF9*, *E2F2*, *SP140*, *DIRC3*, *TNFAIP6*, *LOC349160*, *BAALC*, *VWCE*), tumor suppression (*H19*, *EF2F*) and gametogenesis (*OVOL1*), all of which were with high statistical confidence down-regulated in fibroblasts in comparison to FACS-isolated small putative ovarian stem cells, as can be seen in Additional file 1: Table S5. Similar to hESCs, the putative ovarian stem cells did not express several genes among those which were up-regulated in human fibroblasts (Figure 14).

#### Comparison of all groups of analyzed cells

**Figure 15:**
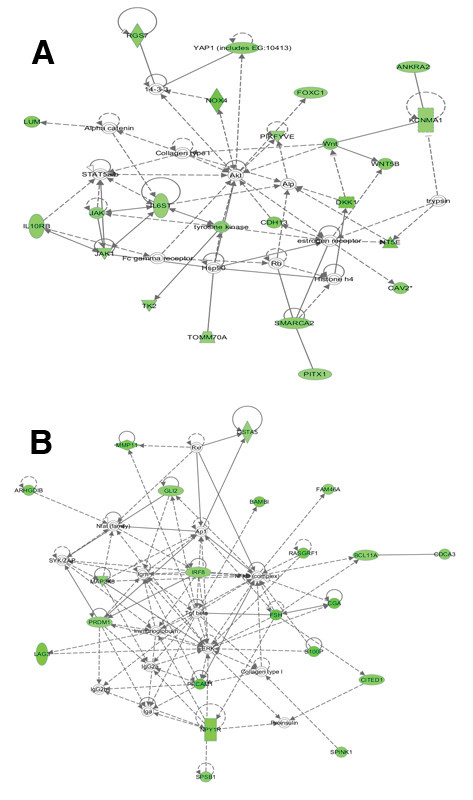
**Gene networks down-regulated in human embryonic stem cells and fibroblasts in comparison with FACS-isolated small putative ovarian stem cells.** (**A**) Network of genes related to organism development, skeletal and muscular system development and function, and embryonic development, which were down-regulated in hESCs. (**B**) Network of genes related to cellular growth and proliferation, organ morphology, and tissue morphology which were down-regulated in human fibroblasts.

The FACS-isolated SSEA-4-positive small putative ovarian stem cells showed lower general gene expression profile than hESCs and fibroblasts which may be related to a more quiescent state. In spite of that, FACS-isolated small putative ovarian stem cells expressed several genes related to pluripotency and embryogenesis which were mostly up-regulated in hESCs. As can been seen in the Additional file 1: Table S6, this reflects in predominating cellular processes, expressed in these two types of cells, such as cell death, molecular transport, cellular growth and proliferation and post-translational modification, as revealed by IPA (Ingenuity) analysis. The fibroblasts did not express or were down-regulated for several genes related to pluripotency and embryogenesis (Figures 12 and 13, Additional file 1: Table S5), and were more related to other cellular processes, such as cell-to-cell signaling and interaction, cellular movement, vitamin and mineral metabolism, antigen presentation and cellular development (Additional file 1: Table S6). Although FACS-isolated SSEA-4-positive small putative ovarian stem cells showed a relatively low general gene expression profile, IPA (Ingenuity) analysis revealed networks of genes related to organism development, skeletal and muscular system development and function, and embryonic development, which were down-regulated in hESCs, as well as networks of genes related to cellular growth and proliferation, organ morphology and tissue morphology which were down-regulated in human fibroblasts, as compared to the OSCs (Figure 15).

In hESCs and small putative ovarian stem cells other transcription regulators were expressed differently (e.g. predominately *MYC*-up-regulated in hESCs, *TP53*-down-regulated-inhibited-in hESCs, *TP63*-up-regulated in hESCs, *AR*-down-regulated in hESCs, *IRF8*-down-regulated in hESCs, *RAR*, *SMAD2/3-SMAD4*, *VITAMIND3-VDR-RXR*, and *NFAT* family), than in somatic fibroblasts and small putative ovarian stem cells (e.g. predominately *SATB1*-inhibited in fibroblasts, *RAR ligand-RARα-Retinoic acid-RXRα*, *RXR*, *PROP1*, *SOX11*, *SOX12*, *E2F7*, *LHX3).*

All this data confirmed the different molecular character of analyzed cells.

### RT-PCR analyses of small putative ovarian stem cells in comparison with hESCs and human adult fibroblasts

**Figure 16:**
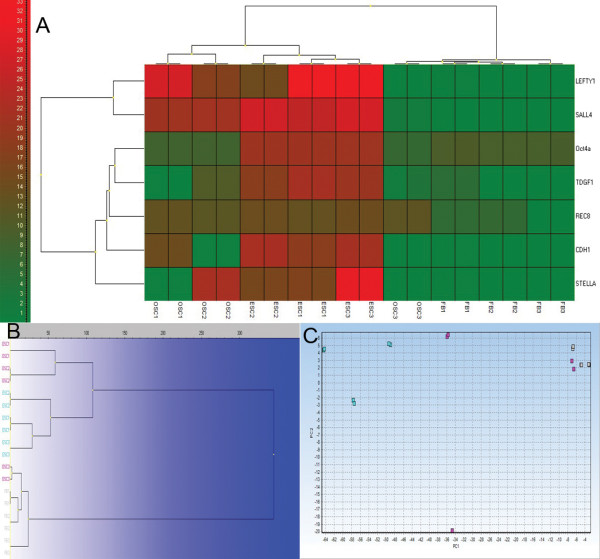
**Small putative ovarian stem cells (OSC1-3: violet) expressed genes related to pluripotency and embryonic stem cells when compared to hESCs (ESC1-3: aquamarine) and human adult (dermal) fibroblasts (FB1-3: gray), as revealed by RT-PCR (Fluidigm).** Two OSC samples clustered with hESCs and one OSC sample clustered with fibroblasts. (**A**) Heatmap clustering; (**B**) Hierarchical clustering; (**C**) Principal component analysis (PCA).

**Figure 17:**
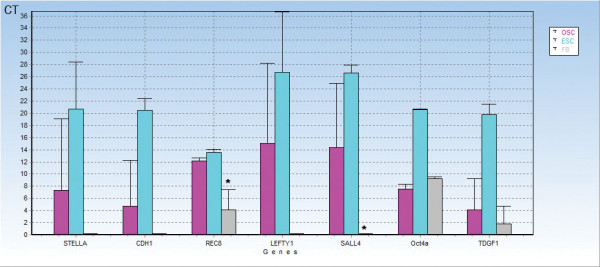
**Descriptive statistics of the expression of genes related to pluripotency and embryonic stem cells by putative ovarian stem cells (OSC1-3: violet) in comparison with hESCs (ESC1-3: aquamarine) and human adult fibroblasts (FB: gray), revealed by RT-PCR (Fluidigm) showing that putative ovarian stem cells and hESCs strongly expressed all analyzed genes, while fibroblasts only weakly expressed them (except*OCT4A*).** In addition, fibroblasts expressed the genes *SALL4* and *REC8* to a significantly lower extent than OSCs, as revealed by the Student’s T-test.

Putative ovarian stem cells and hESCs strongly expressed all analyzed genes (*LEFTY1*, *SALL4*, *OCT4A*, *TDGF1*, *REC8*, *CDH1*, *STELLA*) related to pluripotency and embryonic stem cells, while human adult fibroblasts (FBs) extremely weakly expressed genes *LEFTY1*, *SALL4*, *CDH1* and *STELLA* (Figure 16). In addition, fibroblasts expressed genes *SALL4* (p = 0.0059) and *REC8* (p = 0.0001) to a significantly lower extent than putative ovarian stem cells (OSCs), as revealed by Student’s T-test (Figure 17). Two samples of putative ovarian stem cells (Samples 1 and 2) clustered together with hESCs and were separated from fibroblasts (Figure 16), as revealed by heatmap, dendrogram and principal component analysis. On the other hand, one sample of putative ovarian stem cells (Sample 3) clustered with fibroblasts, which indicates the potential heterogeneity of putative ovarian stem cell samples in terms of pluripotency. These data confirmed the microarray data which similarly showed the up-regulation of genes *LEFTY1*, *SALL4* and *CDH1* in putative ovarian stem cells in comparison with human adult fibroblasts.

## Discussion

We found that in human adult ovarian cell cultures there was a population of small, round, yellow-coloured cells with diameters of up to 4 μm observed during culturing and after isolation by FACS, based on SSEA-4 expression. In testicular and hESC cell cultures there was a similar population of small, round, yellow-coloured SSEA-4-positive cells with diameters of up to 4 μm, isolated by FACS. The expression of SSEA-4 surface antigen makes them putative stem cells. The presence of these small cells – putative stem cells – has already been published by our group for both the human ovarian and testicular cell cultures [18,22], but to our knowledge this is the first time they are reported in hESC cultures.

Small round putative ovarian stem cells may be comparable to VSELs in terms of their morphology, shape and diameters [1], although they seem to be an integral part of the ovaries [18] and not “contaminant” from the blood stream. Surprisingly, these cells were also present and sorted from the hESC cultures. This opens the possible question if these cells are the “real” embryonic stem cells, which may persist in adult human ovaries and testicles from the embryonic period of life – from the time of indifferent gonads. Additionally, this may confirm the potential presence of cells with embryonic-like character in adult human gonads, ovaries and testicles. After FACS-sorting a proportion of small round putative stem cells spontaneously grew into bigger cells with diameters of up to 10 μm.

For ESCs it has been believed that they most closely resemble pluripotent primitive ectoderm cells derived directly from the inner cell mass of blastocysts. However, differences between ESCs and primitive ectoderm cells have opened the question whether ESCs really have an in vivo equivalent or whether their properties mostly reflect their artificial tissue culture environment. In fact, we do not currently know what the natural embryonic stem cells look like.

Microarray data show that FACS-isolated SSEA-4-positive small, round, yellow-coloured cells isolated from the adult ovarian tissue cell cultures express several genes related to adult stem cells, primordial germ cells, pluripotency, embryogenesis, implantation and development; therefore, we propose that they are putative stem cells. In fibroblasts the gene *PROM1* encoding the CD133 surface antigen was significantly down-regulated in comparison with small putative ovarian stem cells. CD133 is known to be expressed in different types of adult stem cells, such as hematopoietic stem cells [28] and in VSELs from human umbilical cord blood [10], and is related to the manifestation of different cancers, including ovarian cancer [29]. Primordial germ cell (PGC)-related genes *PRDM1* (*BLIMP1*) and *PRDM14* were strongly expressed in small putative ovarian stem cells. The *PRDM1* gene is the key determinant of PGCs and plays a crucial role together with *PRDM14* during PGC specification from postimplantation epiblast cells [30]. Moreover, *PRDM1* is a critical determinant of germ cell lineage in mice. The experiments indicated that the PRDM1-positive cells originated from the proximal posterior epiblast cells and were indeed the lineage-restricted PGC precursors [31]. The small putative ovarian stem cells also expressed the *DPPA3* gene. Therefore, the relation of small putative stem cells from the adult ovarian surface epithelium to PGCs or their precursors is not excluded. The microarray data also show a specific pattern of expression of genes related to pluripotency and embryogenesis with several genes down-regulated in comparison with hESCs and others up-regulated in comparison with fibroblasts. In spite of that, these small putative ovarian stem cells show a different, more quiescent gene expression profile than hESCs analyzed in this study. This may reflect the fact that they were isolated from adult ovarian cortex tissues and not from human embryos, and that they passed through the procedure of FACS isolation. It is not excluded that these small putative ovarian stem cells are in a more quiescent stage in comparison with other analyzed cells (hESCs, fibroblasts), as already published for some other types of stem cells, such as hematopoietic stem cells [32] and cancer stem cells [33]. This “dormant” stage may enable them to persist in adult tissues until they are needed for regeneration. Small putative ovarian stem cells showed completely different gene expression profiles than human fibroblasts, which did not express several genes related to pluripotency, embryogenesis and development, or were down-regulated for them. The small size of the putative stem cells from adult gonads (up to 4 μm) comparable to VSELs from human bone marrow or umbilical cord blood with diameters of ~5–6 μm could probably be explained by their highly quiescent status.

It is necessary to expose the down-regulation of epigenetically-regulated genes *H19* and *IGF2* in fibroblasts and the down-regulation of *H19* in hESCs in comparison with small putative ovarian stem cells in this study. This may only be in partial accordance with the previous finding in bone marrow-derived VSELs that confirmed the up-regulation of *H19* and down-regulation of *IGF2* in VSELs [25]. It has been proposed that the up-regulation of *H19* in VSELs might be involved in the maintenance of the quiescent stage and prevention of teratoma formation in adult tissues [25]. Small putative ovarian stem cells expressed a specific pattern of up-regulated genes related to pluripotency and embryonic stem cells, including genes *SALL4*, *LEFTY1*, *CDH1*, *ZIC3* and *HESRG*. The stem cell gene *SALL4* is well characterized for its essential role in developmental events as well as embryonic stem cell pluripotency maintenance. This gene was confirmed to have a critical role in the development of embryonic germ cells and differentiation of postnatal spermatogonial progenitor cells [34]. It is also expressed in hematopoietic stem cells and represents a key regulator in normal human hematopoiesis [35]. This might reflect the same (mesodermal) origin of both the ovaries and circulatory system (including hematopoietic stem cells), and the same progenitor cells; it is not excluded that the progenitor cells may circulate in the blood stream. In addition, the CDH1 (e-cadherin) is known to be the late premeiotic germ cell marker [36]. The character of small putative stem cells in reproductive tissues needs to be further researched to make a real conclusion.

Interestingly, there were several genes related to germinal lineage expressed in both the hESCs and small putative ovarian stem cells. Most of them were up-regulated in hESCs, while the genes *VASA* and *PLD6* were up-regulated in small putative ovarian stem cells. Gene *PLD6* is required for gametogenesis and promotes the recruitment and/or activation of components of the meiotic nuage [37]. Additionally, a proportion of cell colonies and small round cells – putative stem cells – in all cell cultures, regardless of origin, were stained VASA-positive. The gene *VASA* is a germinal lineage-associated gene, which is expressed in human gametes [38]. The VASA-staining was reasonable because the possible germ cell origin of hESCs has already been suggested [39], and it is not excluded that the oocyte represents a real source of “embryonic” stem cells. Little is known about the *VASA* expression and expression of other germinal lineage-related genes in pluripotent stem cells. Some studies revealed the expression of germinal lineage-related genes, including *VASA*, by ESCs in primates [40]. Therefore the germ cell origin of both hESCs and small putative stem cells from reproductive tissues cannot be excluded.

## Competing interests

The authors declare that they have no competing interests.

## Authors’ contributions

IVK contributed to the acquisition, experimental design, analysis and interpretation of the data, drafting the article and the project’s financial support. MS contributed to the analysis and critical revision of the paper. BC and EVB contributed to the recruitment of patients and ovarian tissue retrieval. TS contributed to the analysis, financial support and critical revision of the paper. All authors read and approved the final manuscript.

## Supplementary Material

Additional file 1: Table S1 FACS-isolated, SSEA-4-positive, small putative ovarian stem cells (OSCs) expressed several genes related to pluripotency, cell self-renewal, embryonic development and implantation. All of these genes were up-regulated in hESCs at a high statistical confidence, as revealed by DGA analysis (hESCs vs. OSCs). **Table S2.** FACS-isolated, SSEA-4-positive, small putative ovarian stem cells (OSCs) expressed some germinal lineage-related genes. Most of these genes were up-regulated in hESCs at a high statistical confidence with the exception of genes *DDX4* (*VASA*) and *PLD6,* which were down-regulated in hESCs, as revealed by DGA analysis (hESCs vs. OSCs). Most of these genes were not expressed in human fibroblasts when compared to OSCs. **Table S3.** Genes down-regulated in human embryonic stem cells in comparison with FACS-isolated, SSEA-4-positive, small putative ovarian stem cells at a high statistical confidence. *Legend*: *– genes downregulated in human fibroblasts at a high statistical confidence, **– genes down-regulated in human fibrobalsts at a statistical significance of log ratio < -4. **Table S4.** Genes which were at a high statistical confidence up-regulated in human embryonic stem cells in comparison with FACS-isolated, SSEA-4-positive, small putative ovarian stem cells. **Table S5.** Genes which were at a high statistical confidence down-regulated in human fibroblasts in comparison with FACS-isolated, SSEA-4-positive, small putative ovarian stem cells. **Table S6.** Genes differently expressed in human embryonic stem cells (hESCs) and FACS-isolated, SSEA-4-positive, small putative ovarian stem cells (OSCs) showed different relations to associated network functions, diseases and disorders, molecular and cellular functions, physiological system development and function and top canonical pathways than genes differently expressed in fibroblasts and OSCs. Click here for file
