# Supplementary material for: Small SSEA-4-positive cells from human ovarian cell cultures: related to embryonic stem cells and germinal lineage?
Source: J Ovarian Res. 2013 Apr 9;6:24. doi: 10.1186/1757-2215-6-24 (PMC3660272; doi:10.1186/1757-2215-6-24)
Supplement: Additional file 1: Table S1 — FACS-isolated, SSEA-4-positive, small putative ovarian stem cells (OSCs) expressed several genes related to pluripotency, cell self-renewal, embryonic development and implantation. All of these genes were up-regulated in hESCs at a high statistical confidence, as revealed by DGA analysis (hESCs vs. OSCs). Table S2. FACS-isolated, SSEA-4-positive, small putative ovarian stem cells (OSCs) expressed some germinal lineage-related genes. Most of these genes were up-regulated in hESCs at a high statistical confidence with the exception of genes DDX4 (VASA) and PLD6, which were down-regulated in hESCs, as revealed by DGA analysis (hESCs vs. OSCs). Most of these genes were not expressed in human fibroblasts when compared to OSCs. Table S3. Genes down-regulated in human embryonic stem cells in comparison with FACS-isolated, SSEA-4-positive, small putative ovarian stem cells at a high statistical confidence. Legend: *– genes downregulated in human fibroblasts at a high statistical confidence, **– genes down-regulated in human fibrobalsts at a statistical significance of log ratio < -4. Table S4. Genes which were at a high statistical confidence up-regulated in human embryonic stem cells in comparison with FACS-isolated, SSEA-4-positive, small putative ovarian stem cells. Table S5. Genes which were at a high statistical confidence down-regulated in human fibroblasts in comparison with FACS-isolated, SSEA-4-positive, small putative ovarian stem cells. Table S6. Genes differently expressed in human embryonic stem cells (hESCs) and FACS-isolated, SSEA-4-positive, small putative ovarian stem cells (OSCs) showed different relations to associated network functions, diseases and disorders, molecular and cellular functions, physiological system development and function and top canonical pathways than genes differently expressed in fibroblasts and OSCs. [file 1757-2215-6-24-S1.doc]

**Supplemental Table 1.** FACS-isolated, SSEA-4-positive, small putative ovarian stem cells (OSCs) expressed several genes related to pluripotency, cell self-renewal, embryonic development and implantation. All of these genes were upregulated in hESCs at a high statistical confidence, as revealed by DGA analysis (hESCs vs. OSCs).

**Supplemental Table 2.** FACS-isolated, SSEA-4-positive, small putative ovarian stem cells (OSCs) expressed some germinal lineage-related genes. Most of these genes were upregulated in hESCs at a high statistical confidence with the exception of genes *DDX4* (*VASA*) and *PLD6,* which were down-regulated in hESCs, as revealed by DGA analysis (hESCs vs. OSCs). Most of these genes were not expressed in human fibroblasts when compared to OSCs.

**Supplemental Table 3.** Genes downregulated in human embryonic stem cells in comparison with FACS-isolated, SSEA-4-positive, small putative ovarian stem cells at a high statistical confidence. *Legend*: *– genes downregulated in human fibroblasts at a high statistical confidence, **– genes downregulated in human fibrobalsts at a statistical significance of log ratio < -4.

**Supplemental Table 4.** Genes which were at a high statistical confidence upregulated in human embryonic stem cells in comparison with FACS-isolated, SSEA-4-positive, small putative ovarian stem cells.

**Supplemental Table 5.** Genes which were at a high statistical confidence downregulated in human fibroblasts in comparison with FACS-isolated, SSEA-4-positive, small putative ovarian stem cells.

**Supplemental Table 6.** Genes differently expressed in human embryonic stem cells (hESCs) and FACS-isolated, SSEA-4-positive, small putative ovarian stem cells (OSCs) showed different relations to associated network functions, diseases and disorders, molecular and cellular functions, physiological system development and function and top canonical pathways than genes differently expressed in fibroblasts and OSCs.

Supplemental Table 1

| GENE | GENE NAME | LOCATION | FUNCTION  (Gene Cards) |
| --- | --- | --- | --- |
| ***POU5F1 (OCT4)***  p = 2.99E-06  Fold change  38.691 | POU domain class 5, transcription factor 1 | Chromosome 6 | -a role in embryonic development, especially during early embryogenesis,  -necessary for embryonic stem cell pluripotency. |
| ***SALL4***  p = 8.66E-04  Fold change  16.544 | sal-like 4 (Drosophila) | Chromosome 20 | -a zinc finger transcription factor,  -involved in the formation of tissues and organs during embryonic development,  -plays critical role in limb development, development of nerves that control eye movement, and formation of walls (septa) that divide heart into separate chambers. |
| ***CDH1***  p = 5.21E-03  Fold change  211.736 | cadherin 1, type 1, E-cadherin (epithelial) | Chromosome 16 | -important functions in pluripotency and reprogramming,  -involved in mechanisms regulating cell-cell adhesions, mobility and proliferation of (epithelial) cells,  -loss of function is thought to contribute to progression in cancer (also ovarian) by increasing proliferation, invasion, and/or metastasis. |
| ***LIN28B***  p = 3.07E-02  Fold change  37.059 | lin-28 homolog B  (C. elegans) | Chromosome 6 | -a marker of undifferentiated human embryonic [stem cells](http://en.wikipedia.org/wiki/Stem_cell). |
| ***NANOG***  p = 1.24E-02  Fold change  135.621 | Nanog homeobox | Chromosome 12 | -expressed in embryonic stem and cells carcinoma cells,  -expressed in inner cell mass of the blastocyst and gonocytes between 14 and 19 weeks of gestation,  -not expressed in oocytes, unfertilized oocytes, 2-16 cell embryos and early morula,  -expression decreases with differentiation of embryonic stem cells. |
| ***SOX2***  p = 1.09E-03  Fold change  414.986 | SRY (sex determining region Y)-box 2 | Chromosome 3 | -controls the expression of a number of genes involved in embryonic development such as *YES1*, *FGF4*, *UTF1* and *ZFP206*,  -critical for early embryogenesis and for embryonic stem cell pluripotency,  -functions as a switch in neuronal development. Keeps neural cells undifferentiated by counteracting the activity of proneural proteins and suppresses neuronal differentiation. |
| ***SOX11***  p = 7.89E-03  Fold change  31.209 | SRY (sex determining region Y)-box 11 | Chromosome 2 | -transcription factor involved in the regulation of embryonic development and in the determination of the cell fate, -acts as a transcriptional regulator after forming a protein complex with other proteins,  -functions in the developing nervous system and plays a role in tumorigenesis. |
| ***DPPA3 (STELLA)***  p = 2.79E-07  Fold change  114.680 | developmental pluripotency associated 3 | Chromosome 12 | -may play a role in maintaining cell pluripotency. |
| ***LEFTY1***  p = 1.72E-02  Fold change  53.455 | left-right determination factor 1 | Chromosome 1 | -plays a role in left-right asymmetry determination of organ systems during development. |
| ***ZIC3***  p = 2.52E-03  Fold change  320.468 | Zic family member 3 | Chromosome X | -acts as transcriptional activator,  -required in the earliest stages of development in both axial midline development and left-right (LR) asymmetry specification,  -mutations cause X-linked visceral heterotaxy, which includes congenital heart disease and left-right axis defects in organs. |
| ***ZIC5***  p = 5.36E-03  Fold change  99.837 | Zic family member 5 | Chromosome 13 | -encodes a member of the ZIC family of C2H2-type zinc finger proteins,  -important during development,  -associated with X-linked visceral heterotaxy and holoprosencephaly type 5,  -essential for neural crest development, converting cells from an epidermal fate to a neural crest cell fate. |
| ***PRDM14***  p = 6.41E-03  Fold change  65.066 | PR domain containing 14 | Chromosome 8 | -involved in transcriptional regulation in human embryonic stem cells,  -involved in the maintenance of the self-renewal of human embryonic stem cells [34],  -suppress the expression of genes related to differentiation. |
| ***GAL***  p = 1.30E-02  Fold change  34.949 | galanin prepropeptide | Chromosome 11 | -found in proliferating zones of the fetal and adult brain and in peripheral tissues including the pancreas, heart, skin and gastrointestinal tract. |
| ***PPP1R9A***  p = 8.28E-03  Fold change  22.383 | protein phosphatase 1, regulatory (inhibitor) subunit 9A | Chromosome 7 | -this gene is imprinted, and located in a cluster of imprinted genes on chromosome 7q12,  -transcribed in both neuronal and multiple embryonic tissues,  -maternally expressed mainly in embryonic skeletal muscle tissues and biallelically expressed in other embryonic tissues. |
| ***RNF2***  p = 2.41E-03  Fold change  142.565 | ring finger protein 2 | Chromosome 1 | -polycomb group (PcG) of proteins form the multiprotein complexes important for the transcription repression of various genes involved in development and cell proliferation,  -involvement of this gene in the specification of anterior-posterior axis, as well as in cell proliferation in early development. |
| ***LASS1 (CERS1)***  p = 7.09E-03  Fold change  125.356 | ceramide synthase 1 | Chromosome 19 | -encodes a member of the bone morphogenetic protein (BMP) family and the TGF-beta superfamily,  -members of this family are regulators of cell growth and differentiation in both embryonic and adult tissues,  -encoded protein is involved in aging. |
| ***SMO***  p = 4.97E-03  Fold change  58.046 | smoothened, frizzled family receptor | Chromosome 7 | -the protein encoded by this gene is a G protein-coupled receptor that interacts with the patched protein, a receptor for hedgehog proteins.  -Smo receptors are vital for embryogenesis (segmentation and appendage development), -involved in maintenance of tissue homeostasis in adults. |
| ***MMP25***  p = 3.94E-03  Fold change  146.813 | matrix metallopeptidase 25 | Chromosome 16 | -proteins of the matrix metalloproteinase (MMP) family are involved in the breakdown of extracellular matrix in normal physiological processes, such as embryonic development, reproduction, and tissue remodeling, as well as in disease processes, such as arthritis and metastasis. |
| ***GULP 1***  p = 3.37E-02  Fold change  17.998 | GULP, engulfment adaptor PTB domain containing 1 | Chromosome 2 | -the prompt clearance of cells undergoing apoptosis is critical during embryonic development, normal tissue turnover, inflammation, and autoimmunity. |
| ***MLLT4***  p = 4.85E-03  Fold change  59.773 | myeloid/lymphoid or mixed-lineage leukemia (trithorax homolog, Drosophila); translocated to, 4 | Chromosome 6 | -encodes a multi-domain protein involved in signaling and organization of cell junctions during embryogenesis,  -identified as the fusion partner of acute lymphoblastic leukemia (ALL-1) gene, involved in acute myeloid leukemias. |
| ***BMP7***  p = 7.15E-03  Fold change  95.102 | bone morphogenetic protein 7 | Chromosome 20 | -induces cartilage and bone formation,  -based on its expression early in embryogenesis, the BMP encoded by this gene has a proposed role in early development and possible bone inductive activity. |
| ***MYBL2***  p = 7.29E-04  Fold change  22.380 | v-myb myeloblastosis viral oncogene homolog (avian)-like 2 | Chromosome 20 | -the protein encoded by this gene, a member of the MYB family of transcription factor genes, is a nuclear protein involved in cell cycle progression,  -transcription factor involved in the regulation of cell survival, proliferation, and differentiation. |
| ***DNMT3B***  p = 3.07E-03  Fold change  106.878 | DNA (cytosine-5-)-methyltransferase 3 beta | Chromosome 20 | -highly expressed in embryonic stem cells,  -encodes DNA methyltransferase responsible for unmethylated CpG island methylation. CpG methylation is an epigenetic modification that is important for embryonic development, imprinting, and X-chromosome inactivation,  -required for mammalian development. |
| ***ZFP42***  p = 1.88E-04  Fold change  485.281 | zinc finger protein 42 homolog (mouse) | Chromosome 4 | -involved in self-renewal property of embryonic stem cells,  -may be involved in transcriptional regulation. |
| ***HESRG***  p = 3.65E-02  Fold change  67.149 | Human embryonic stem cells related gene | Chromosome 3 | -highly expressed in undifferentiated human embryonic stem cells,  -it maintains the pluripotency state and self-renewal. |
| ***ZSCAN10***  p = 3.49E-03  Fold change  23.597 | zinc finger and SCAN domain containing 10 | Chromosome 16 | -embryonic stem cell-specific transcription factor required to maintain cell pluripotency. |
| ***TRO***  p = 2.61E-02  Fold change  32.869 | trophinin | Chromosome X | -encodes a membrane protein that mediates apical cell adhesion between trophoblastic cells and luminal epithelial cells of the endometrium,  -implicated in the initial attachment during the process of embryo implantation. |
| ***GLI2***  p = 1.68E-02  Fold change  23.652 | GLI family zinc finger 2 | Chromosome 2 | -acts as a transcriptional activator,  -may play a role during embryogenesis. |
| ***FBN3***  p = 1.53E-03  Fold change  158.808 | fibrillin 3 | Chromosome 19 | -most highly expressed in fetal tissues,  -its protein product is localized to extracellular microfibrils of developing skeletal elements, skin, lung, kidney, and skeletal muscle. |
| ***DDX11***  p = 5.22E-03  Fold change  74.511 | DEAD/H (Asp-Glu-Ala-Asp/His) box polypeptide 11 | Chromosome 12 | -DEAD box proteins, characterized by the conserved motif Asp-Glu-Ala-Asp (DEAD), are putative RNA helicases,  -based on their distribution  patterns, some members of this family are believed to be involved in embryogenesis, spermatogenesis, and cellular  growth and division. |

**Supplemental Table 2**

| GENE | GENE NAME | LOCATION | FUNCTION  (Gene Cards) |
| --- | --- | --- | --- |
| ***DDX4 (VASA)***  p = 9.68E-03  Fold change  -319.821 | DEAD (Asp-Glu-Ala-Asp) box polypeptide 4 | Chromosome 5 | -involved in embryogenesis, spermatogenesis, and cellular growth and division,  -specifically expressed in the germ cell lineage in both sexes and functions in germ cell development. |
| ***DPPA3 (STELLA)***  p = 2.79E-07  Fold change  114.680 | developmental pluripotency associated 3 | Chromosome 12 | -may play a role in maintaining cell pluripotency,  -germ cell marker. |
| ***KIT***  p = 2.96E-02  Fold change 42.718 | v-kit Hardy-Zuckerman 4 feline sarcoma viral oncogene homolog | Chromosome 4 | -encodes the human homolog of the proto-oncogene c-kit,  -the protein is a type 3 transmembrane receptor for mast cell growth factor, also known as stem cell factor and KIT ligand,  -promotion of primordial follicle activation, oocyte growth, and follicle survival [35],  -mutations in this gene are associated with cancer. |
| ***DIAPH2***  p = 3.44E-03  Fold change  74.087 | diaphanous homolog 2 (Drosophila) | Chromosome X | -may play a role in the development and normal function of the ovaries, defects in this gene have been linked to premature ovarian failure 2,  -could be involved in oogenesis. |
| ***NR6A1***  p = 3.64E-07  Fold change  557.763 | nuclear receptor subfamily 6, group A, member 1 | Chromosome 9 | -this gene encodes an orphan nuclear receptor which is a member of the nuclear hormone receptor family,  -its expression pattern suggests that it may be involved in neurogenesis and germ cell development. |
| ***PLD6***  p = 2.38E-02  Fold change  -18.036 | phospholipase D family, member 6 | Chromosome 17 | -the production of phosphatidate regulates the piRNA metabolic process by promoting recruitment and/or activation of components of the meiotic nuage, also named P granule, a critical step for primary biogenesis of piRNAs,  -required during gametogenesis to repress transposable elements and prevent their mobilization via its role in the piRNA metabolic process. |
| ***DNMT3B***  p = 3.07E-03  Fold change  106.878 | DNA (cytosine-5-)-methyltransferase 3 beta | Chromosome 20 | -highly expressed in embryonic stem cells,  -encodes DNA methyltransferase responsible for unmethylated CpG island methylation. CpG methylation is an epigenetic modification that is important for embryonic development, imprinting, and X-chromosome inactivation,  -required for mammalian development. |
| ***BNC2***  p = 5.21E-01  Fold change 23.987 | basonuclin 2 | Chromosome 9 | -transcription factor,  -plays a role  in the differentiation of  oocytes and sperm |
| ***MSH4***  p = 2.30E-01  Fold change 21.312 | mutS homolog 4  (E. coli) | Chromosome 1 | -involved in meiotic recombination,  -required for reciprocal recombination and proper segregation of homologous  chromosomes at meiosis |

**Supplemental Table 3**

| GENE | GENE NAME | LOCATION | FUNCTION  (Gene Cards) |
| --- | --- | --- | --- |
| ***CGA****  p = 5.22E-03  Fold change  -42.406 | Glycoprotein hormones, alpha polypeptide | Chromosome 6 | -the protein is the alpha subunit of four human glycoprotein hormones: chorionic gonadotropin (CG), luteinizing hormone (LH), follicle stimulating hormone (FSH), and thyroid stimulating hormone (TSH). |
| ***FRMPD4****  p = 1.03E-02  Fold change  -23.550 | FERM and PDZ domain containing 4 | Chromosome X | -positive regulator of dendritic spine morphogenesis and density. |
| ***TGM2****  p = 1.82E-02  Fold change  -16.952 | transglutaminase 2 (C polypeptide, protein-glutamine-gamma-glutamyltransferase) | Chromosome 20 | -involved in apoptosis. |
| ***S100P***  p = 5.11E-05  Fold change  -23.767 | S100 calcium binding protein P | Chromosome 4 | -involved in the regulation of a number of cellular processes such as cell cycle progression and differentiation  -involved in the etiology of the prostate cancer. |
| ***HOXD9***  p = 1.97E-03  Fold change  -126.830 | homeobox D9 | Chromosome 2 | -important role in morphogenesis in all multicellular organisms,  -mutations associated with severe limb and genital abnormalities,  -provides cells with specific positional identities on the anterior-posterior axis. |
| ***PECAM1****  p = 1.73E-03  Fold change  -70.239 | platelet/endothelial cell adhesion molecule | Chromosome 17 | -involved in leukocyte migration, angiogenesis, and integrin activation. |
| ***PITX1****  p = 1.53E-02  Fold change  -16.859 | paired-like homeodomain 1 | Chromosome 5 | -involved in organ development (in particular, the brain and facies) and left-right asymmetry,  -transcriptional regulator involved in basal and hormone-regulated activity of prolactin. |
| ***ARHGDIB****  p = 1.47E-04  Fold change  -63.561 | Rho GDP dissociation inhibitor (GDI) beta | Chromosome 12 | -involved in diverse cellular events, including cell signaling, proliferation, cytoskeletal organization and secretion. |
| ***NOX4****  p = 1.05E-03  Fold change  -90.564 | NADPH oxidase 4 | Chromosome 11 | -the reactive oxygen species generated by this protein implicated in numerous biological functions including signal transduction, cell differentiation and tumor cell growth. |
| ***FRY****  p = 3.87E-03  Fold change  -57.888 | furry homolog (Drosophila) | Chromosome 13 | -plays a key role in maintaining the integrity of polarized cell extensions during morphogenesis,  -regulates the actin cytoskeleton,  -plays a key role in patterning sensory neuron dendritic fields by promoting avoidance between homologous dendrites as well as by limiting dendritic branching,  -may function as a transcriptional activator. |
| ***FOXL2***  p = 1.45E-04  Fold change  -35.812 | forkhead box L2 | Chromosome 3 | -encodes a forkhead transcription factor,  -the protein contains a fork-head DNA-binding domain,  -plays a role in ovarian development and function,  -mutations in this gene are related to blepharophimosis syndrome and premature ovarian failure. |
| ***H19*****  p = 1.25E-01  Fold change  -10.573 | H19, imprinted maternally expressed transcript (non-protein coding) | Chromosome 11 | -expresses a non-coding RNA,  -functions as a tumor suppressor,  -located in an imprinted region of chromosome 11 near the insulin-like growth factor 2 (IGF2) gene,  -the expression of this gene and IGF2 are imprinted so that this gene is only expressed from the maternally-inherited chromosome, and IGF2 is only expressed from the paternally-inherited chromosome,  -a region of paternal-specific methylation upstream of this gene is required for the imprinting of these genes,  -mutations in this gene are associated with Beckwith-Wiedemann Syndrome and Wilms  tumorigenesis. |
| ***PSG5***  p =1.87E-03  Fold change  -67.345 | pregnancy specific beta-1-glycoprotein 5 | Chromosome 19 | -encodes human pregnancy-specific glycoprotein, a molecule that is mainly produced by the placental  syncytiotrophoblasts during pregnancy. |
| ***MPP1***  p = 1.89E-02  Fold change  -27.530 | membrane protein, palmitoylated 1, 55kDa | Chromosome X | -interacts with the cytoskeleton,  -regulates cell proliferation, signaling pathways, and intercellular junctions,  -interacts with various cytoskeletal and junctional proteins in different tissue and cell types,  -may be involved in the regulation of cell shape, hair cell development, neural patterning of the retina, and apico-basal polarity,  -tumor suppression pathways. |
| ***WNT5B***  p = 1.01E-04  Fold change  -30.317 | wingless-type MMTV integration site family, member 5B | Chromosome 12 | -implicated in oncogenesis,  -several developmental processes, including regulation of cell fate and  patterning during embryogenesis. |
| ***SPDYA***  p = 3.40E-02  Fold change  -35.861 | speedy homolog A (Xenopus laevis) | Chromosome 2 | -regulates the G1/S phase transition of the cell cycle,  -mediates cell survival during the DNA damage process. |
| ***HOXA11****  p = 2.35E-02  Fold change  -20.385 | homeobox A11 | Chromosome 7 | -sequence-specific transcription factor,  -a part of developmental regulatory system that provides cells with specific positional identities on the anterior-posterior axis,  -involved in the regulation of uterine development and is required for female fertility. |
| ***PPAPDC3***  p = 1.80E-02  Fold change  -119.240 | phosphatidic acid phosphatase type 2 domain containing 3 | Chromosome 9 | -plays a role as negative regulator of myoblast differentiation. |
| ***SMARCA2***  p = 1.10E-02  Fold change  -23.007 | SWI/SNF related, matrix associated, actin dependent regulator of chromatin, subfamily a, member 2 | Chromosome 9 | -belongs to the neural progenitors,  -during neural development enables  a switch from a stem/progenitor to a post-mitotic chromatin remodeling mechanism, which occurs as neurons exit the cell cycle and become committed to their adult state,  -the transition from proliferating neural stem/progenitor cells to post-mitotic neurons. |
| ***NT5E***  p = 9.39E-03  Fold change  -64.955 | 5'-nucleotidase, ecto (CD73) | Chromosome 6 | -determinant of lymphocyte  differentiation. |
| ***BRIP1***  p = 5.88E-03  Fold change  -16.567 | BRCA1 interacting protein C-terminal helicase 1 | Chromosome 17 | -the protein encoded by this gene is a member of the RecQ DEAH helicase family and interacts with the BRCT repeats of breast cancer, type 1 (BRCA1),  -this gene may be a target of germline cancer-inducing mutations. |
| ***CDK8***  p = 9.44E-03  Fold change  -22.991 | cyclin-dependent kinase 8 | Chromosome 13 | -the protein encoded by this gene is an important regulator of cell cycle progression. |
| ***SERPINB8***  p = 2.82E-02  Fold change  -34.371 | serpin peptidase inhibitor, clade B (ovalbumin), member 8 | Chromosome 18 | -encodes a member of the superfamily of serpins which regulate processes, such as complement activation, fibrinolysis, coagulation, cellular differentiation, tumor suppression, apoptosis, and cell migration. |
| ***FOXC1***  p = 3.78E-03  Fold change  -19.471 | forkhead box C1 | Chromosome 6 | -transcription factor,  -it plays a role in the regulation of embryonic and ocular development. |
| ***PML***  p = 6.58E-04  Fold change  -18.344 | promyelocytic leukemia | Chromosome 15 | -functions as a transcription factor and tumor suppressor,  -regulates the response to oncogenic signals and plays a role in regulation of cell division, terminal differentiation of myeloid precursor cells and neural progenitor cells, in neoangiogenesis, tumor vascularization.  -required for normal development of the brain cortex during embryogenesis. |
| ***DAAM1***  p = 2.44E-02  Fold change  -47.142 | dishevelled associated activator of morphogenesis 1 | Chromosome 14 | -implicated in functions of the cell cortex, including motility, adhesion, and cytokinesis,  -controls the cell polarity and movement during development. |
| ***FOXO4***  p = 3.48E-03  Fold change  -86.759 | forkhead box O4 | Chromosome X | -transcription factor,  -involved in the regulation of the insulin signaling pathway,  -involved in growth and differentiation,  -involved in negative regulation of the cell cycle. |
| ***NRP2***  p = 9.52E-03  Fold change  -44.848 | neuropilin 2 | Chromosome 2 | -the protein may play a role in cardiovascular development, axon guidance and tumorigenesis. |
| ***TGFB2***  p = 1.14E-03  Fold change  -22.281 | transforming growth factor, beta 2 | Chromosome 1 | -encodes a member of the transforming growth factor beta (TGFB) family of cytokines, which are multifunctional peptides that regulate proliferation, differentiation, adhesion, migration, and other functions in many cell types. |
| ***IGFBP5***  p = 1.84E-02  Fold change  -63.985  ***IGFBP3***  p = 4.31E-03  Fold change  -35.458 | insulin-like growth factor binding protein 5  insulin-like growth factor binding protein 3 | Chromosome 2  Chromosome 7 | -IGF-binding proteins prolong the half-life of the IGFs and have been shown to either inhibit or stimulate the growth promoting effects of the IGFs on cell culture. |
| ***CDH13***  p = 1.01E-02  Fold change  -33.106 | cadherin 13, H-cadherin (heart) | Chromosome 16 | -encodes a member of the cadherin superfamily,  -acts as a negative regulator of axon growth during neural differentiation, -protects vascular endothelial cells from apoptosis due to oxidative stress,  -acts as a negative regulator of neural cell growth,  -hypermethylated in many types of cancer. |
| ***RUNX1***  p = 2.80E-02  Fold change  -33.727 | runt-related transcription factor 1 | Chromosome 21 | -involved in the development of normal hematopoiesis. |
| ***MR1***  p = 1.66E-02  Fold change  -40.439 | major histocompatibility complex, class I-related | Chromosome 1 | -antigen presentation function,  -involved in the development and expansion of a small population of T cells expressing an invariant T cell receptor alpha chain called mucosal-associated invariant T cells (MAIT). |
| ***CFLAR***  p = 4.84E-03  Fold change  -75.890 | CASP8 and FADD-like apoptosis regulator | Chromosome 2 | -apoptosis regulator protein which functions as a crucial link between cell survival and cell death pathways in mammalian cells. |
| ***CCNY***    p = 7.62E-05  Fold change  -192.679 | cyclin Y | Chromosome 10 | -cyclins, such as CCNY, control cell division cycles. |
| ***FAM114A1***  p = 1.34E-02  Fold change  -17.518 | family with sequence similarity 114, member A1 | Chromosome 4 | -a role in neuronal cell development. |
| ***SPON2***  p = 4.32E-02  Fold change  -49.874 | spondin 2, extracellular matrix protein | Chromosome 4 | -cell adhesion protein that promotes adhesion and outgrowth of hippocampal embryonic neurons. |
| ***CPA4***  p = 2.30E-02  Fold change  -22.433 | carboxypeptidase A4 | Chromosome 7 | -imprinted gene,  -a strong candidate gene for prostate cancer aggressiveness. |
| ***ABLIM3***  p = 8.38E-04  Fold change  -106.857 | actin binding LIM protein family, member 3 | Chromosome 5 | -plays a role in embryonic development,  -cell lineage determination,  -manifestation of cancer. |
| ***CSNK1G3***  p = 9.15E-05  Fold change  -18.881 | casein kinase 1, gamma 3 | Chromosome 5 | -casein kinase I (CK1) is a monomeric serine-threonine protein kinase,  -involved in a number of cellular processes including DNA repair, cell division, nuclear localization and membrane transport,  -has a key role in the developmentally important Wnt and Hedgehog (Hh) signaling pathways. |
| ***ANGPT1***  p = 5.24E-04  Fold change  -28.301 | angiopoietin 1 | Chromosome 8 | -angiopoietin protein has an important role in vascular development and angiogenesis,  -may play an important role in the heart early development. |
| ZBTB26  p = 1.95E-03  Fold change  -20.971 | zinc finger and BTB domain containing 26 | Chromosome 9 | -involved in transcriptional regulation. |
| ***HMBOX1***  p = 1.32E-02  Fold change  -48.675 | homeobox containing 1 | Chromosome 8 | -transcription factor,  -isoform 1 acts as a transcriptional repressor. |
| ***DLEU2L***  p = 7.58E-03  Fold change  -23.213 | deleted in lymphocytic leukemia 2-like | Chromosome 1 | -may act as a tumor suppressor. |
| ***KIAA1324***  p = 2.62E-02  Fold changes  -18.745 | KIAA1324 | Chromosome 1 | -plays a role as a marker of hyperestrogenic state and estrogen-related type I endometrial carcinoma. |
| ***PLXNB3***  p = 1.05E-02  Fold change  -20.056 | plexin B3 | Chromosome X | -the protein is a member of the plexin family,  -functions as a receptor for semaphorin 5A,  -plays a role in axon guidance, invasive growth and cell migration. |
| ***MGLL***  p = 4.97E-02  Fold change  -56.972 | monoglyceride lipase | Chromosome 3 | -regulates the levels of fatty acids that serve as signaling molecules,  -promotes cancer cell migration, invasion and tumor growth. |
| ***ZNF41***  p = 3.24E-03  Fold change  -23.190 | zinc finger protein 41 | Chromosome X | -involved in transcriptional regulation. |
| ***STAM***  p = 1.78E-03  Fold change  -24.709 | signal transducing adaptor molecule (SH3 domain and ITAM motif) 1 | Chromosome 10 | -plays a role in T-cell development. |
| ***DYRK4***  p = 7.39E-03  Fold change  -19.733 | dual-specificity tyrosine-(Y)-phosphorylation regulated kinase 4 | Chromosome 12 | -dual-specificity kinases, such as DYRK4,  -plays key roles in cell proliferation, survival, and development. |
| ***PARVA***  p = 4.56E-03  Fold change  -41.729 | parvin, alpha | Chromosome 11 | -the protein is part of the integrin-linked kinase signaling complex,  -plays a role in cell adhesion, motility and survival,  -probably plays a role in the regulation of cell adhesion and cytoskeleton organization,  -plays a role in ciliogenesis. |
| ***PHTF2***  p = 4.95E-02  Fold change  -21.169 | putative homeodomain transcription factor 2 | Chromosome 7 | -may play a role in transcription regulation. |
| ***MXI1***  p = 1.32E-03  Fold change  -26.183 | MAX interactor 1 | Chromosome 10 | -expression of the c-myc gene, which produces an oncogenic transcription factor, is tightly regulated in normal cells but is frequently deregulated in human cancers,  -the protein encoded by this gene is a transcriptional repressor thought to negatively regulate MYC function, and is therefore a potential tumor suppressor. |
| ***YAP1***  p = 2.30E-02  Fold change  -21.488 | Yes-associated protein 1 | Chromosome 11 | -transcriptional regulator which can act both as a coactivator and a corepressor,  -is the critical downstream regulatory target that plays a pivotal role in organ size control and tumor suppression by restricting proliferation and promoting apoptosis,  -plays a key role to control cell proliferation in response to cell contact,  -phosphorylation of YAP1 regulates cellular genes important for cell proliferation, cell death, and cell migration. |
| ***PCDHGB4***  p = 1.04E-02  Fold change  -101.618 | protocadherin gamma subfamily B, 4 | Chromosome 5 | -involved in the establishment and maintenance of specific neuronal connections in the brain. |
| ***MBD2***  p = 1.03E-02  Fold change  -20.043 | methyl-CpG binding domain protein 2 | Chromosome 18 | -the protein may function as a mediator of the biological consequences of the methylation signal. Binds CpG islands in promoters where the DNA is methylated at position 5 of cytosine within CpG dinucleotides,  -acts as transcriptional repressor and plays a role in gene silencing. |
| ***PLD6***  p = 2.38E-02  Fold change  -18.036 | phospholipase D family, member 6 | Chromosome 17 | -the production of phosphatidate regulates the piRNA metabolic process by promoting recruitment and/or activation of components of the meiotic nuage, also named P granule, a critical step for primary biogenesis of piRNAs,  -required during gametogenesis to repress transposable elements and prevent their mobilization via its role in the piRNA metabolic process. |
| ***ZNF518B***  p = 1.14E-02  Fold change  -17.533 | zinc finger protein 518B | Chromosome 4 | -transcriptional factor. |
| ***ZNF703***  p = 3.38E-03  Fold change  -40.609 | zinc finger protein 703 | Chromosome 8 | -regulates cell adhesion, migration and proliferation,  -may be required for segmental gene expression during hindbrain development. |
| ***LUM***  p = 1.11E-02  Fold change  -34.102 | Lumican | Chromosome 12 | -lumican may regulate collagen fibril organization and circumferential growth, corneal transparency, and epithelial cell migration and tissue repair. |
| ***SEPT8***  p = 1.19E-02  Fold change  -27.128 | septin 8 | Chromosome 5 | -it is a member of the highly conserved septin family,  -involved in the organization of submembranous structures, in neuronal polarity, and in vesicle  trafficking,  -plays a role in cytokinesis. |
| ***INTS6***  p = 4.49E-02  Fold change  -20.693 | integrator complex subunit 6 | Chromosome 13 | -may have a tumor suppressor role,  -an ectopic expression suppresses tumor cell growth. |
| ***FBLN5***  p = 4.45E-02  Fold change  -35.158 | fibulin 5 | Chromosome 14 | -plays a role in vascular development and remodeling. |
| ***CACNA1C***  p = 8.97E-04  Fold change  -37.986 | calcium channel, voltage-dependent, L type, alpha 1C subunit | Chromosome 12 | -voltage-sensitive calcium channels (VSCC) mediate a variety of calcium-dependent processes, including muscle contraction, hormone or neurotransmitter release, gene expression, cell motility, cell division and cell death. |
| ***SEMA5A***  p = 4.92E-03  Fold change  -17.937 | sema domain, seven thrombospondin repeats (type 1 and type 1-like), transmembrane domain (TM) and short cytoplasmic domain, (semaphorin) 5A | Chromosome 5 | -involved in axonal guidance during neural development. |
| ***ACVR1***  p = 4.61E-05  Fold change  -52.983 | activin A receptor, type I | Chromosome 2 | -involved for left-right pattern formation during embryogenesis. |
| ***XIST***  p = 4.11E-03  Fold change  -49.149 | X (inactive)-specific transcript (non-protein coding) | Chromosome X | -X inactivation is an early developmental process in mammalian females that transcriptionally silences one of the pair of X chromosomes, thus providing dosage equivalence between males and females. The process is regulated by several factors, including a region of chromosome X called the X inactivation center (XIC). The XIST gene is expressed exclusively from the XIC of the inactive X chromosome. |
| ***FGF7***  p = 3.97E-02  Fold change  -20.237 | fibroblast growth factor 7 | Chromosome 15 | -FGF family members possess broad mitogenic and cell survival activities,  -involved in a variety of biological processes, including embryonic development, cell growth, morphogenesis, tissue repair, tumor growth and invasion,  -a potent epithelial cell-specific growth factor, whose mitogenic activity is predominantly exhibited in keratinocytes but not in fibroblasts and endothelial cells,  -important in morphogenesis  of epithelium, reepithelialization of wounds, hair development and early lung organogenesis. |
| ***UACA***  p = 2.68E-04  Fold change  -19.228 | uveal autoantigen with coiled-coil domains and ankyrin repeats | Chromosome 15 | -plays an important role in the regulation of stress-induced apoptosis,  -regulates the morphological alterations required for cell growth and motility,  -interaction with ARF6 may modulate cell shape and motility after injury. |
| ***GPNMB***  p = 8.46E-04  Fold change  -158.302 | glycoprotein (transmembrane) nmb | Chromosome 7 | -involved in growth delay and reduction of metastatic potential. |
| ***IL6ST***  p = 1.33E-03  Fold change  -18.777 | interleukin 6 signal transducer (gp130, oncostatin M receptor) | Chromosome 5 | -signal-transducing molecule,  -may have a role in embryonic development. |
| ***TTC8***  p = 1.15E-04  Fold change  -28.689 | tetratricopeptide repeat domain 8 | Chromosome 14 | -this gene is expressed in ciliated cells and is involved in the formation of cilia. |
| ***CAV2***  p = 1.33E-04  Fold change  -25.905 | caveolin 2 | Chromosome 7 | -the protein is a major component of the inner surface of caveolae, small invaginations of the plasma membrane,  -involved in essential cellular functions, including signal transduction, lipid metabolism, cellular growth control and apoptosis  -may function as a tumor suppressor. |
| ***MPP4***  p = 1.19E-02  Fold change  -17.988 | membrane protein, palmitoylated 4 (MAGUK p55 subfamily member 4) | Chromosome 2 | -plays a role in retinal photoreceptors development. |
| ***PPM1F***  p = 2.29E-03  Fold change  -16.881 | protein phosphatase, Mg2+/Mn2+ dependent, 1F | Chromosome 22 | -promotes apoptosis. |

Supplemental Table 4

| GENE | GENE NAME | LOCATION | FUNCTION  (Gene Cards) |
| --- | --- | --- | --- |
| ***SALL4***  p = 8.66E-04  Fold change  16.544 | sal-like 4 (Drosophila) | Chromosome 20 | -a zinc finger transcription factor,  -involved in the formation of tissues and organs during embryonic development,  -plays critical role in limb development, development of nerves that control eye movement, and formation of walls (septa) that divide heart into separate chambers. |
| ***POU5F1 (OCT4)***  p = 2.99E-06  Fold change  38.691 | POU domain class 5, transcription factor 1 | Chromosome 6 | -a role in embryonic development, especially during early embryogenesis,  -necessary for embryonic stem cell pluripotency. |
| ***ZFP42***  p = 1.88E-04  Fold change  485.281 | zinc finger protein 42 homolog (mouse) | Chromosome 4 | -involved in self-renewal property of embryonic stem cells,  -may be involved in transcriptional regulation. |
| ***PIM1***  p = 7.60E-03  Fold change  227.536 | pim-1 oncogene | Chromosome 6 | -contributes to both cell proliferation and survival,  -provides a selective advantage in tumorigenesis. |
| ***DMKN***  p = 8.67E-03  Fold change  913.207 | dermokine | Chromosome 19 | -this gene is upregulated in inflammatory diseases and was first found to be expressed in the differentiated layers of skin,  -may act as a soluble regulator of keratinocyte differentiation. |
| ***CDH1***  p = 5.21E-03  Fold change  211.736 | cadherin 1, type 1, E-cadherin (epithelial) | Chromosome 16 | -involved in mechanisms regulating cell-cell adhesions, mobility and proliferation of (epithelial) cells,  -loss of function is thought to contribute to progression in cancer (also ovarian) by increasing proliferation, invasion, and/or metastasis. |
| ***LIN28B***  p = 3.07E-02  Fold change  37.059 | lin-28 homolog B  (C. elegans) | Chromosome 6 | -a marker of undifferentiated human embryonic [stem cells](http://en.wikipedia.org/wiki/Stem_cell). |
| ***NANOG***  p = 1.24E-02  Fold change  135.621 | Nanog homeobox | Chromosome 12 | -expressed in embryonic stem and cells carcinoma cells,  -expressed in inner cell mass of the blastocyst and gonocytes between 14 and 19 weeks of gestation,  -not expressed in oocytes, unfertilized oocytes, 2-16 cell embryos and early morula,  -expression decreases with differentiation of embryonic stem cells. |
| ***SOX2***  p = 1.09E-03  Fold change  414.986 | SRY (sex determining region Y)-box 2 | Chromosome 3 | -controls the expression of a number of genes involved in embryonic development such as YES1, FGF4, UTF1 and ZFP206,  -critical for early embryogenesis and for embryonic stem cell pluripotency,  -functions as a switch in neuronal development. Keeps neural cells undifferentiated by counteracting the activity of proneural proteins and suppresses neuronal differentiation. |
| ***SOX11***  p = 7.89E-03  Fold change  31.209 | SRY (sex determining region Y)-box 11 | Chromosome 2 | -transcription factor involved in the regulation of embryonic development and in the determination of the cell fate, -acts as a transcriptional regulator after forming a protein complex with other proteins,  -functions in the developing nervous system and plays a role in tumorigenesis. |
| ***LEFTY1***  p = 1.72E-02  Fold change  53.455 | left-right determination factor 1 | Chromosome 1 | -plays a role in left-right asymmetry determination of organ systems during development. |
| ***LECT1***  p = 2.23E-04  Fold change  18.097 | leukocyte cell derived chemotaxin 1 | Chromosome 13 | -promotes chondrocyte growth and inhibits angiogenesis,  -involved in the control of tissue vascularization during development,  -bifunctional growth regulator that stimulates the growth of cultured chondrocytes but inhibits the growth of vascular endothelial cells, -contributes to the rapid growth of cartilage and vascular invasion prior to the replacement of cartilage by bone during bone development. |
| ***ZIC3***  p = 2.52E-03  Fold change  320.468 | Zic family member 3 | Chromosome X | -acts as transcriptional activator,  -required in the earliest stages of development in both axial midline development and left-right (LR) asymmetry specification,  -mutations cause X-linked visceral heterotaxy, which includes congenital heart disease and left-right axis defects in organs. |
| ***ZIC 5***  p = 5.36E-03  Fold change  99.837 | Zic family member 5 | Chromosome 13 | -encodes a member of the ZIC family of C2H2-type zinc finger proteins,  -important during development,  -associated with X-linked visceral heterotaxy and holoprosencephaly type 5,  -essential for neural crest development, converting cells from an epidermal fate to a neural crest cell fate. |
| ***CLDN6***  p = 5.97E-04  Fold change  203.125 | claudin 6 | Chromosome 16 | -plays a major role in tight junction-specific obliteration of the intercellular space in epithelial or endothelial cell sheets,  -the gene methylation may be involved in tumorigenesis. |
| ***CLDN7***  p = 3.15E-02  Fold change  17.019 | Claudin 7 | Chromosome 17 | -plays a major role in tight junction-specific obliteration of the intercellular space in epithelial or endothelial cell sheets,  -differential expression of this gene has been observed in different types of malignancies, including breast and ovarian cancer. |
| ***CLDN10***  p = 4.96E-02  Fold change  23.518 | claudin 10 | Chromosome 13 | -plays a major role in tight junction-specific obliteration of the intercellular space in epithelial or endothelial cell sheets,  -the expression level is associated with recurrence of primary hepatocellular carcinoma. |
| ***CECR2***  p = 2.16E-04  Fold change  1001.210 | cat eye syndrome chromosome region, candidate 2 | Chromosome 22 | -facilitates the perturbation of chromatin structure,  -may be involved in the integration of cytoskeletal network with vesicular trafficking, nucleocytosolic shuttling, transcription, chromosome remodeling, cytokinesis. |
| ***SFRP2***  p = 1.63E-03  Fold change  128.887 | secreted frizzled-related protein 2 | Chromosome 4 | -modulator of Wnt signaling through direct interaction with Wnts,  -regulates cell growth and differentiation in specific cell types,  -may be important for eye retinal development and for myogenesis,  -methylation of this gene is related to cancer. |
| ***GDPD2***  p = 7.35E-04  Fold change  706.721 | glycerophosphodiester phosphodiesterase domain containing 2 | Chromosome X | -the encoded protein hydrolyzes glycerophosphoinositol to produce inositol 1-phosphate and glycerol,  -may play a role in remodeling of the actin cytoskeleton and osteoblast  differentiation and growth. |
| ***PRDM14***  p = 6.41E-03  Fold change  65.066 | PR domain containing 14 | Chromosome 8 | -involved in transcriptional regulation. |
| ***GAL***  p = 1.30E-02  Fold change  34.949 | galanin prepropeptide | Chromosome 11 | -found in proliferating zones of the fetal and adult brain and in peripheral tissues including the pancreas, heart, skin and gastrointestinal tract. |
| ***ANP32E***  p = 9.83E-04  Fold change  476.987 | cidic (leucine-rich) nuclear phosphoprotein 32 family, member E | Chromosome 1 | -may play a role in cerebellar development and synaptogenesis process by modulating PP2A activity. |
| ***ZNF721***  p = 1.81E-02  Fold change  68.170 | zinc finger protein 721 | Chromosome 4 | -may be involved in transcriptional regulation. |
| ***ZNF75D***  p = 5.17E-03  Fold change  27.508 | zinc finger protein 75D | Chromosome X | -may be involved in transcriptional regulation. |
| ***ZFP64***  p = 2.52E-03  Fold change  181.804 | zinc finger protein 64 homolog (mouse) | Chromosome 20 | -may be involved in transcriptional regulation. |
| ***ZNF845***  p = 4.63E-02  Fold change  24.943 | zinc finger protein 845 | Chromosome 19 | -may be involved in transcriptional regulation. |
| ***ZNF48***  p = 3.91E-02  Fold change  18.003 | zinc finger protein 48 | Chromosome 16 | -may be involved in transcriptional regulation. |
| ***ZNF766***  p = 1.71E-02  Fold change  40.793 | zinc finger protein 766 | Chromosome 19 | -may be involved in transcriptional regulation. |
| ***RBM38***  p = 3.27E-03  Fold change  166.959 | RNA binding motif protein 38 | Chromosome 20 | -it has the ability to induce cell cycle arrest in G1. Plays a role in myogenic differentiation |
| ***EPB41L5***  p = 1.74E-03  Fold change  47.198 | erythrocyte membrane protein band 4.1 like 5 | Chromosome 2 | -may contribute to the correct positioning of tight junctions during the establishment of polarity in epithelial cells. |
| ***MYO5A***  p = 5.63E-06  Fold change  156.795 | myosin VA (heavy chain 12, myoxin) | Chromosome 15 | -may also be required for some polarization process involved in dendrite formation. |
| ***BEX1***  p = 3.54E-05  Fold change  238.913 | brain expressed, X-linked 1 | Chromosome X | -plays a role in cell cycle progression and neuronal differentiation. |
| ***GPM6B***  p = 3.52E-02  Fold change  25.620 | glycoprotein M6B | Chromosome X | -may be involved in neural development. |
| ***SASH1***  p = 1.53E-03  Fold change  234.196 | SAM and SH3 domain containing 1 | Chromosome 6 | -may have a role in a signaling pathway,  -could act as a tumor suppressor. |
| ***OLFM1***  p = 1.47E-04  Fold change  481.422 | olfactomedin 1 | Chromosome 9 | -seems to play an important role in regulating the production of neural crest cells by the neural tube. |
| ***DIAPH2***  p = 3.44E-03  Fold change  74.087 | diaphanous homolog 2 (Drosophila) | Chromosome X | -may play a role in the development and normal function of the ovaries, defects in this gene have been linked to premature ovarian failure 2,  -could be involved in oogenesis. |
| ***ROR1***  p = 3.45E-02  Fold change  32.967 | receptor tyrosine kinase-like orphan receptor 1 | Chromosome 1 | -the protein is a receptor protein tyrosine kinase that modulates neurite growth in the central nervous system. |
| ***NAV2***  p = 4.87E-03  Fold change  122.751 | neuron navigator 2 | Chromosome 11 | -plays an important role in neuronal development, including neurite outgrowth. Involved in neuronal development, specifically in the development of different sensory organs. |
| ***USP2***  p = 1.63E-02  Fold change  18.603 | ubiquitin specific peptidase 2 | Chromosome 11 | -plays a role in the G1/S cell-cycle progression in normal and cancer cells,  -plays a role in the regulation of myogenic differentiation of embryonic muscle cells. |
| ***RNF38***  p = 2.41E-03  Fold change  142.565 | ring finger protein 38 | Chromosome 9 | -the RING motif is a zinc-binding domain found in a large set of proteins playing roles in diverse cellular processes including oncogenesis, development, signal transduction, and apoptosis. |
| ***SNRPN***  p = 7.29E-04  Fold change  134.553 | Small nuclear ribonucleoprotein polypeptide N | Chromosome 15 | -the protein plays a role in pre-mRNA processing, possibly tissue-specific alternative splicing events. The 5' UTR of this gene has been identified as an imprinting center. Alternative splicing or deletion caused by a translocation event in this paternally-expressed region is responsible for Angelman syndrome or Prader-Willi syndrome due to parental imprint switch failure. |
| ***ACTR3C***  p = 2.62E-02  Fold change  87.677 | ARP3 actin-related protein 3 homolog C (yeast) | Chromosome 7 | -may play a role in the suppression of metastatic potential in lung adenoma carcinoma cells. |
| ***MAP7***  p = 6.51E-03  Fold change  120.561 | microtubule-associated protein 7 | Chromosome 6 | -microtubule-stabilizing protein that may play an important role during reorganization of microtubules during polarization and differentiation of epithelial cells. |
| ***PPP1R9A***  p = 8.28E-03  Fold change  22.383 | protein phosphatase 1, regulatory (inhibitor) subunit 9A | Chromosome 7 | -this gene is imprinted, and located in a cluster of imprinted genes on chromosome 7q12,  -transcribed in both neuronal and multiple embryonic tissues,  -maternally expressed mainly in embryonic skeletal muscle tissues and biallelically expressed in other embryonic tissues. |
| ***CAPN12***  p = 2.01E-02  Fold change  58.723 | calpain 12 | Chromosome 19 | -calpains are a family of cytosolic calcium-activated cysteine proteases involved in a variety of cellular processes including apoptosis, cell division, modulation of integrin-cytoskeletal interactions, and synaptic plasticity. |
| ***WWC1***  p = 1.95E-02  Fold change  18.541 | WW and C2 domain containing 1 | Chromosome 5 | -probable regulator of the Hippo/SWH (Sav/Wts/Hpo) signaling pathway, a signaling pathway that plays a pivotal  role in tumor suppression by restricting proliferation and promoting apoptosis. |
| ***CEP164***  p = 3.37E-03  Fold change  26.142 | centrosomal protein 164kDa | Chromosome 11 | -plays a role in microtubule organization and/or maintenance for the formation of primary cilia (PC), a microtubule-based structure that protrudes from the surface of pithelial cells,  -plays a critical role in G2/M checkpoint and nuclear divisions. |
| ***BCL9***  p = 6.69E-03  Fold change  34.034 | B-cell CLL/lymphoma 9 | Chromosome 1 | -BCL9 is associated with B-cell acute lymphoblastic leukemia,  -it may be a target of translocation in B-cell malignancies with abnormalities of 1q21,  -the overexpression of BCL9 may be of pathogenic significance in B-cell malignancies. |
| ***RNF2***  p = 2.41E-03  Fold change  142.565 | ring finger protein 2 | Chromosome 1 | -polycomb group (PcG) of proteins form the multiprotein complexes important for the transcription repression of various genes involved in development and cell proliferation,  -involvement of this gene in the specification of anterior-posterior axis, as well as in cell proliferation in early development. |
| ***LASS1 (CERS1)***  p = 7.09E-03  Fold change  125.356 | ceramide synthase 1 | Chromosome 19 | -encodes a member of the bone morphogenetic protein (BMP) family and the TGF-beta superfamily,  -members of this family are regulators of cell growth and differentiation in both embryonic and adult tissues,  -encoded protein is involved in aging. |
| ***DCLK1***  p = 7.87E-04  Fold change  208.592 | doublecortin-like kinase 1 | Chromosome 13 | -the encoded protein is involved in several different cellular processes,  including neuronal migration, retrograde transport, neuronal apoptosis and neurogenesis. |
| ***SMO***  p = 4.97E-03  Fold change  58.046 | smoothened, frizzled family receptor | Chromosome 7 | -the protein encoded by this gene is a G protein-coupled receptor that interacts with the patched protein, a receptor for hedgehog proteins.  -Smo receptors are vital for embryogenesis (segmentation and appendage development), -involved in maintenance of tissue homeostasis in adults. |
| ***PIM2***  p = 1.28E-02  Fold change  229.575 | pim-2 oncogene | Chromosome X | -encodes a protooncogene that acts as a serine/threonine protein kinase,  -the encoded protein functions to prevent apoptosis and to promote cell survival. |
| ***SPINT2***  p = 6.77E-03  Fold change  70.421 | serine peptidase inhibitor, Kunitz type, 2 | Chromosome 19 | -this gene is a putative tumor suppressor. |
| ***MMP25***  p = 3.94E-03  Fold change  146.813 | matrix metallopeptidase 25 | Chromosome 16 | -proteins of the matrix metalloproteinase (MMP) family are involved in the breakdown of extracellular matrix in normal physiological processes, such as embryonic development, reproduction, and tissue remodeling, as well as in disease processes, such as arthritis and metastasis. |
| ***WDR92***  p = 9.96E-03  Fold change  224.799 | WD repeat domain 92 | Chromosome 2 | -a diverse range of functions, including signal transduction, cell cycle regulation, RNA splicing, and transcription. |
| ***GULP 1***  p = 3.37E-02  Fold change  17.998 | GULP, engulfment adaptor PTB domain containing 1 | Chromosome 2 | -the prompt clearance of cells undergoing apoptosis is critical during embryonic development, normal tissue turnover, inflammation, and autoimmunity. |
| ***MLLT4***  p = 4.85E-03  Fold change  59.773 | myeloid/lymphoid or mixed-lineage leukemia (trithorax homolog, Drosophila); translocated to, 4 | Chromosome 6 | -encodes a multi-domain protein involved in signaling and organization of cell junctions during embryogenesis,  -identified as the fusion partner of acute lymphoblastic leukemia (ALL-1) gene, involved in acute myeloid leukemias. |
| ***NR6A1***  p = 3.64E-07  Fold change  557.763 | nuclear receptor subfamily 6, group A, member 1 | Chromosome 9 | -this gene encodes an orphan nuclear receptor which is a member of the nuclear hormone receptor family,  -its expression pattern suggests that it may be involved in neurogenesis and germ cell development. |
| ***SBK1***  p = 1.40E-02  Fold change  54.386 | SH3-binding domain kinase 1 | Chromosome 16 | -may be involved in signal-transduction pathways related to the control of brain development. |
| ***BMP7***  p = 7.15E-03  Fold change  95.102 | bone morphogenetic protein 7 | Chromosome 20 | -induces cartilage and bone formation,  -based on its expression early in embryogenesis, the BMP encoded by this gene has a proposed role in early development and possible bone inductive activity. |
| ***S1PR5***  p = 1.79E-03  Fold change  54.462 | sphingosine-1-phosphate receptor 5 | Chromosome 19 | -the lysosphingolipid sphingosine 1-phosphate (S1P) regulates cell proliferation, apoptosis, motility, and neurite retraction,  -may play a regulatory role in the transformation of radial glial cells into astrocytes,  -may affect proliferative activity of these cells. |
| ***ITM2C***  p = 5.75E-03  Fold change  39.227 | integral membrane protein 2C | Chromosome 2 | -may play a role in TNF-induced cell death and neuronal differentiation. |
| ***IFI6***  p = 3.18E-03  Fold change  264.017 | interferon, alpha-inducible protein 6 | Chromosome 1 | -may play a critical role in the regulation of apoptosis. |
| ***SFRP2***  p = 1.63E-03  Fold change  128.887 | secreted frizzled-related protein 2 | Chromosome 4 | -modulator of Wnt signaling, -methylation of this gene is a potential marker for the presence of colorectal cancer, -a role in regulating cell growth and differentiation in specific cell types,  -may be important for eye retinal development and for myogenesis. |
| ***MYBL2***  p = 7.29E-04  Fold change  22.380 | v-myb myeloblastosis viral oncogene homolog (avian)-like 2 | Chromosome 20 | -the protein encoded by this gene, a member of the MYB family of transcription factor genes, is a nuclear protein involved in cell cycle progression,  -transcription factor involved in the regulation of cell survival, proliferation, and differentiation. |
| ***ITGB1BP3***  p = 3.90E-02  Fold change  39.089 | integrin beta 1 binding protein 3 | Chromosome 19 | -may play a role in the regulation of terminal myogenesis. |
| ***DNMT3B***  p = 3.07E-03  Fold change  106.878 | DNA (cytosine-5-)-methyltransferase 3 beta | Chromosome 20 | -highly expressed in embryonic stem cells,  -encodes DNA methyltransferase responsible for unmethylated CpG island methylation. CpG methylation is an epigenetic modification that is important for embryonic development, imprinting, and X-chromosome inactivation,  -required for mammalian development. |
| ***PLXDC1***  p = 1.41E-04  Fold change  30.514 | plexin domain containing 1 | Chromosome 17 | -plays a critical role in endothelial cell capillary morphogenesis. |
| ***NLGN3***  p = 1.73E-02  Fold change  23.904 | neuroligin 3 | Chromosome X | -neuronal cell surface protein thought to be involved in cell-cell-interactions by forming intercellular  junctions through binding to beta-neurexins,  -may play a role in formation or maintenance of synaptic junctions,  -may be involved in the formation and remodeling of central nervous system synapses,  -mutations in this gene may be associated with autism and Asperger syndrome,  -may also play a role in glia-glia or glia-neuron interactions in the developing peripheral nervous system. |
| ***IGSF9***  p = 1.29E-02  Fold change  22.555 | **i**mmunoglobulin superfamily, member 9 | Chromosome 1 | -functions in dendrite outgrowth and synapse maturation. |
| ***SPIB***  p = 2.64E-02  Fold change  40.159 | Spi-B transcription factor (Spi-1/PU.1 related) | Chromosome 19 | -promotes development of plasmacytoid dendritic cells,  -may be required for B-cell receptor (BCR) signaling, which is necessary for normal B-cell development and antigenic stimulation. |
| ***PCDHB14***  p = 4.37E-02  Fold change  42.869 | protocadherin beta 14 | Chromosome 5 | -potential calcium-dependent cell-adhesion protein,  -may be involved in the establishment and maintenance of specific neuronal connections in the brain. |
| ***PCDH21***  p = 1.93E-03  Fold change  52.366 | protocadherin 21 | Chromosome 10 | -a possible role in the formation and maintenance of neuronal networks. |
| ***DPPA3 (STELLA)***  p = 2.79E-07  Fold change  114.680 | developmental pluripotency associated 3 | Chromosome 12 | -may play a role in maintaining cell pluripotentiality. |
| ***UBE2C***  p = 2.31E-03  Fold change  19.186 | ubiquitin-conjugating enzyme E2C | Chromosome 20 | -this enzyme is required for the destruction of mitotic cyclins and for cell cycle progression. |
| ***ZFP42***  p = 1.88E-04  Fold change  485.281 | zinc finger protein 42 homolog (mouse) | Chromosome 4 | -involved in self-renewal property of embryonic stem cells,  -may be involved in transcriptional regulation. |
| ***TRIM71***  p = 1.51E-02  Fold change  94.064 | tripartite motif containing 71 | Chromosome 3 | -may be involved in controlling the timing of organ formation during development. |
| ***HESRG***  p = 3.65E-02  Fold change  67.149 | Human embryonic stem cells related gene | Chromosome 3 | -highly expressed in undifferentiated human embryonic stem cells,  -it maintains the pluripotency state and self-renewal. |
| ***ZSCAN10***  p = 3.49E-03  Fold change  23.597 | zinc finger and SCAN domain containing 10 | Chromosome 16 | -embryonic stem cell-specific transcription factor required to maintain cell pluripotency. |
| ***RCOR2***  p = 2.22E-03  Fold change  30.329 | REST corepressor 2 | Chromosome 11 | -may act as a component of a corepressor complex that represses transcription. |
| ***TRO***  p = 2.61E-02  Fold change  32.869 | trophinin | Chromosome X | -encodes a membrane protein that mediates apical cell adhesion between trophoblastic cells and luminal epithelial cells of the endometrium,  -implicated in the initial attachment during the process of embryo implantation. |
| ***GLI2***  p = 1.68E-02  Fold change  23.652 | GLI family zinc finger 2 | Chromosome 2 | -acts as a transcriptional activator,  -may play a role during embryogenesis. |
| ***FBN3***  p = 1.53E-03  Fold change  158.808 | fibrillin 3 | Chromosome 19 | -most highly expressed in fetal tissues,  -its protein product is localized to extracellular microfibrils of developing skeletal elements, skin, lung, kidney, and skeletal muscle. |
| ***DDX11***  p = 5.22E-03  Fold change  74.511 | DEAD/H (Asp-Glu-Ala-Asp/His) box polypeptide 11 | Chromosome 12 | -DEAD box proteins, characterized by the conserved motif Asp-Glu-Ala-Asp (DEAD), are putative RNA helicases,  -based on their distribution  patterns, some members of this family are believed to be involved in embryogenesis, spermatogenesis, and cellular  growth and division. |
| ***LINGO1***  p = 2.99E-02  Fold change  94.053 | leucine rich repeat and Ig domain containing **1** | Chromosome 15 | -responsible for some inhibition of axonal regeneration by myelin-associated factors,  -an important negative regulator of oligodentrocyte differentiation and axonal myelination. |
| ***CNTNAP2***  p = 2.08E-03  Fold change  29.699 | contactin associated protein-like 2 | Chromosome 7 | -the protein is localized at the  juxtaparanodes of myelinated axons, and mediates interactions between neurons and glia during nervous system development,  -is involved in localization of potassium channels within differentiating axons. |
| ***NOTCH3***  p = 4.26E-03  Fold change  42.794 | notch 3 | Chromosome 19 | -Notch interaction with its cell-bound ligands (delta, serrate) establishes an intercellular signaling pathway that plays a key role in neural development,  -affects the implementation of differentiation, proliferation and apoptotic programs. |
| ***NEUROD1***  p = 4.30E-03  Fold change  26.643 | neurogenic differentiation 1 | Chromosome 2 | -acts as a transcriptional activator,  -contributes to the regulation of several cell differentiation pathways, like those that promote the formation of early retinal ganglion cells, inner ear sensory neurons, granule cells forming either the cerebellum or the dentate gyrus cell layer of the hippocampus, endocrine islet cells of the pancreas and enteroendocrine cells of the small intestine. |
| ***SEMA5B***  p = 2.20E-02  Fold change  89.634 | sema domain, seven thrombospondin repeats (type 1 and type 1-like), transmembrane domain (TM) and short cytoplasmic domain, (semaphorin) 5B | Chromosome 3 | -members of the semaphorin protein family, such as SEMA5B, are involved in axonal guidance during neural development. |
| ***CENPM***  p = 4.70E-02  Fold change  16.556 | centromere protein M | Chromosome 22 | -plays a central role in assembly of kinetochore proteins, mitotic progression and chromosome segregation. |
| ***TUBB2B***  p = 2.42E-03  Fold change  43.666 | tubulin, beta 2B | Chromosome 6 | -the protein is a beta isoform of tubulin, which binds GTP and is a major component of microtubules. Microtubules also form the spindle fibers for separating chromosomes during mitosis. |
| ***MAP2K6***  p = 1.18E-02  Fold change  50.002 | mitogen-activated protein kinase kinase 6 | Chromosome 17 | -involved in many cellular processes such as stress induced cell cycle arrest, transcription activation and apoptosis. |
| ***CTSL2***  p = 2.43E-04  Fold change  53.065 | cathepsin L2 | Chromosome 9 | -expressed in colorectal and breast carcinomas but not in normal colon, mammary gland, or peritumoral tissues, suggesting a possible role for this gene in tumor processes,  -involved in a range of physiological and pathological processes, including maturation of the  MHC class II complex, bone remodeling, keratinocyte differentiation, tumor progression and metastasis,  rheumatoid arthritis, osteoarthritis and atherosclerosis. |
| ***GAP43***  p = 5.93E-04  Fold change  217.679 | growth associated protein 43 | Chromosome 3 | -the protein has been termed a 'growth' or 'plasticity' protein because it is expressed at high  levels in neuronal growth cones during development and axonal regeneration. This protein is considered a crucial  component of an effective regenerative response in the nervous system. |
| ***DIDO1***  P = 4.39E-03  Fold change  42.045 | death inducer-obliterator 1 | Chromosome 20 | -putative transcription factor, weakly pro-apoptotic when overexpressed (By similarity), -tumor suppressor. |

**Supplemental Table 5**

| GENE | GENE NAME | LOCATION | FUNCTION  (Gene Cards) |
| --- | --- | --- | --- |
| ***PITX1***  p = 2.20E-02  Fold change  -18.239 | paired-like homeodomain 1 | Chromosome 5 | -involved in organ development (in particular, the brain and facies) and left-right asymmetry,  -transcriptional regulator involved in basal and hormone-regulated activity of prolactin. |
| ***SALL4***  p = 2.55E-03  Fold change  -31.677 | sal-like 4 (Drosophila) | Chromosome 20 | -a zinc finger transcription factor,  -involved in the formation of tissues and organs during embryonic development,  -plays critical role in limb development, development of nerves that control eye movement, and formation of walls (septa) that divide heart into separate chambers. |
| ***SOX15***  p = 2.96E-04  Fold change  -88.741 | SRY (sex determining region Y)-box 15 | Chromosome 17 | -transcription regulator,  -binds to the 5'-AACAAT-3' sequence,  -involved in the regulation of embryonic development and in the determination of the cell fate. |
| ***ELF3***  p = 5.17E-03  Fold change  -50.308 | E74-like factor 3 (ets domain transcription factor, epithelial-specific) | Chromosome 1 | -transcriptional activator,  -plays an important role in the regulation of transcription with TATA-less promoters in preimplantation embryos, which is essential in preimplantation development,  -may play an important role in epithelial cell differentiation,  -plays an important role in tumorigenesis,  - may be associated with mammary gland development and involution. |
| ***HOXD11***  p = 3.78E-02  Fold change  -40.617 | Homeobox D11 | Chromosome 2 | -the homeobox genes encode a highly conserved family of transcription factors that play an important role in morphogenesis in all multicellular organisms,  -deletions that remove the entire *HOXD* gene cluster or the 5' end of this cluster have been associated with severe limb and genital abnormalities,  –a part of developmental regulatory system that provides cells with specific positional identities on the anterior-posterior axis. |
| ***H19***  p = 4.27E-02  Fold change  -23.653 | H19, imprinted maternally expressed transcript (non-protein coding) | Chromosome 11 | -expresses a non-coding RNA,  -functions as a tumor suppressor,  -located in an imprinted region of chromosome 11 near the insulin-like growth factor 2 (IGF2) gene, -the expression of this gene and IGF2 are imprinted so that this gene is only expressed from the maternally-inherited chromosome, and IGF2 is only expressed from the paternally-inherited chromosome,  -a region of paternal-specific methylation upstream of this gene is required for the imprinting of these genes,  -mutations in this gene are associated with Beckwith-Wiedemann Syndrome and Wilms  tumorigenesis. |
| ***IGF2***  p = 3.47E-02  Fold change  -58.359 | insulin-like growth factor 2 (somatomedin A) | Chromosome 11 | -encodes a member of the insulin family of polypeptide growth factors, which are involved in development and growth,  -it is an imprinted gene, expressed only from the paternal allele, and epigenetic changes at this locus are  associated with Wilms tumour, Beckwith-Wiedemann syndrome, rhabdomyosarcoma, and Silver-Russell syndrome,  -potent mitogens for cultured cells,  -is influenced by placental lactogen and may play a role in fetal development,  -exhibits osteogenic properties by increasing osteoblast mitogenic activity. |
| ***CDCA3***  p = 2.29E-04  Fold change  -22.990 | cell division cycle associated 3 | Chromosome 12 | -protein which is required for entry into mitosis,  -acts by participating to E3 ligase complexes that mediate the ubiquitination and degradation of WEE1 kinase at G2/M phase. |
| ***CENPM***  p = 4.13E-03  Fold change  -28.188 | centromere protein M | Chromosome 22 | -the protein is a component of the CENPA-NAC (nucleosome-associated) complex, that plays a central role in assembly of kinetochore proteins, mitotic progression and chromosome segregation. |
| ***DTL***  p = 3.02E-04  Fold change  -37.677 | denticleless homolog (Drosophila) | Chromosome 1 | -required for cell cycle control, DNA damage response and translesion DNA synthesis,  -important in proliferating cells. |
| ***NOX4***  p = 3.23E-03  Fold change  -62.317 | NADPH oxidase 4 | Chromosome 11 | -the ROS generated by this protein have been implicated in numerous biological functions including signal transduction, cell differentiation and tumor cell growth. |
| ***FGF9***  p = 4.70E-02  Fold change  -16.891 | fibroblast growth factor 9 (glia-activating factor) | Chromosome 13 | -FGF family members possess broad mitogenic and cell survival activities,  -involved in a variety of biological processes, including embryonic development, cell growth, morphogenesis, tissue repair, tumor growth and invasion,  -has a role in glial cell growth and differentiation during development, gliosis during repair and regeneration of brain tissue after damage, differentiation and survival of neuronal cells, and growth stimulation of glial tumors. |
| ***OVOL1***  p = 2.92E-03  Fold change  -32.760 | ovo-like 1(Drosophila**)** | Chromosome 11 | -putative transcription factor,  -the ovo protein may play a critical role in oogenesis, spermatogenesis, hair formation, in the differentiation and/or maintenance of the urogenital system. |
| ***CGA***  p = 1.17E-03  Fold change  -79.408 | glycoprotein hormones, alpha polypeptide | Chromosome 6 | -the protein is the alpha subunit of four human glycoprotein hormones: chorionic gonadotropin (CG), luteinizing hormone (LH), follicle stimulating hormone (FSH), and thyroid stimulating hormone (TSH), which regulate developmental processes. |
| ***ESRRB***  p = 3.31E-06  Fold change  -19.610 | estrogen-related receptor beta | Chromosome 14 | -encodes a protein with similarity to the estrogen receptor. Its function is unknown; a similar protein in mouse plays an essential role in placental development,  -estrogen controls many cellular processes including growth, differentiation and function of the reproductive system. In females, estrogen's main targets are the ovaries, uterus, vagina and mammary glands. |
| ***ZNF114***  p = 8.37E-04  Fold change  -39.182 | zinc finger protein 114 | Chromosome 19 | -may be involved in transcriptional regulation. |
| ***E2F2***  p = 1.44E-03  Fold change  -30.037 | E2F transcription factor 2 | Chromosome 1 | -transcription regulator,  -plays a crucial role in the control of cell cycle,  -the control of cell-cycle progression from g1 to s phase,  -acts as tumor suppressor protein,  -a target of the transforming  proteins of small DNA tumor viruses. |
| ***SP140***  p = 2.76E-05  Fold change  -31.677 | SP140 nuclear body protein | Chromosome 2 | -component of the nuclear body,  -involved in the pathogenesis of acute promyelocytic leukemia. |
| ***DIRC3***  p = 6.13E-05  Fold change  -30.221 | disrupted in renal carcinoma 3 | Chromosome 2 | -disrupted in renal carcinoma 3 |
| ***TNFAIP6***  p = 1.99E-02  Fold change  -20.345 | tumor necrosis factor, alpha-induced protein 6 | Chromosome 2 | -possibly involved in cell-cell and cell-matrix interactions during tumorigenesis |
| ***LOC349160***  p = 4.71E-04  Fold change  -21.332 | Homo sapiens clone N1 NTera2D1 teratocarcinoma mRNA | Chromosome 7 | -involved in tumorigenesis |
| ***BAALC***  p = 3.80E-02  Fold change  -20.363 | brain and acute leukemia, cytoplasmic | Chromosome 8 | -the expression was identified in patients with acute myeloid leukemia (AML),  -some of the transcript variants are found only in AML cell lines. |
| ***VWCE***  p = 1.09E-04  Fold change  -179.395 | von Willebrand factor C and EGF domains | Chromosome 11 | -may be a regulatory element in the beta-catenin signaling pathway,  -a target for chemoprevention of  hapatocellular carcinoma. |

**Supplemental Table S6**

hESCs,OSCs FIB, OSCs

**Associated Network Functions**

Lipid Metabolism, Small Molecule Biochemistry, Carbohydrate Metabolism 37

Inflammatory Response, Cellular Movement, Hematological System Development and Function 35

Cellular Development, Hematopoiesis, Cell Signaling 32

Cell Death, Cellular Growth and Proliferation, Connective Tissue Development and Function 27

Cellular Growth and Proliferation, Cellular Development, Gene Expression 27

Cellular Growth and Proliferation, Cell Death, Molecular Transport 46

Cellular Development, Cell Cycle, Cell Death 31

Amino Acid Metabolism, Small Molecule Biochemistry, Cancer 23

Cell Morphology, Skeletal and Muscular System Development and Function, Tissue Development 20

Cellular Development, Cellular Growth and Proliferation, Hematological System Development 16

and Function

**Diseases and Disorders**

Cancer 93 18

Immunological Disease 57 5

Gastrointestinal Disease 89

Cardiovascular Disease 58

Endocrine System Disorders 53

Inflammatory Response 15

Hematological Disease 10

Developmental Disorder 7

**Molecular and Cellular Functions**

Gene Expression 58

Cell Death 64

Molecular Transport 17

Cellular Growth and Proliferation 65

Post-Translational Modification 10

Cell-To-Cell Signaling and Interaction 13

Cellular Movement 20

Vitamin and Mineral Metabolism 6

Antigen Presentation 5

Cellular Development 15

**Physiological System Development and Function**

Organismal Development 44

Tissue Development 57 20

Cardiovascular System Development and Function 31

Embryonic Development 34 17

Reproductive System Development and Function 11 10

Nervous System Development and Function 11

**Top Canonical Pathways**

Factors Promoting Cardiogenesis in Vertebrates 5/95

Wnt/β-catenin Signaling 7/174

Oncostatin M Signaling 3/35

Extrinsic Prothrombin Activation Pathway 2/20

RAR Activation 6/187

Aminophosphonate Metabolism 2/57

Breast Cancer Regulation by Stathmin 1 4/210 4-3-3-mediated Signaling 3/124
